# Supplementary material for: Region-Specific Impact of Repeated Synthetic Cannabinoid Exposure and Withdrawal on Endocannabinoid Signaling, Gliosis, and Inflammatory Markers in the Prefrontal Cortex and Hippocampus
Source: Biomolecules. 2025 Mar 14;15(3):417. doi: 10.3390/biom15030417 (PMC11940679; doi:10.3390/biom15030417)

Table S1. List of abbreviations for endocannabinoid system, gliosis and inflammation-related genes, their protein products, and corresponding functions.

Gene	Protein	Full Name / Function
Cnr1	CB1	Cannabinoid receptor type 1 – Mainly found in the central nervous system, involved in modulating neurotransmitter release.
Cnr2	CB2	Cannabinoid receptor type 2 – Primarily expressed in immune cells, involved in immune regulation and inflammation.
Ppara	PPARα	Peroxisome proliferator-activated receptor alpha – Regulates lipid metabolism and inflammation.
Nape-pld	NAPE-PLD	N-acyl phosphatidylethanolamine phospholipase D – Key enzyme in the biosynthesis of N-acylethanolamines, including anandamide (AEA).
Daglα	DAGLα	Diacylglycerol lipase alpha – Enzyme responsible for the biosynthesis of 2-arachidonoylglycerol (2-AG), an endocannabinoid.
Daglβ	DAGLβ	Diacylglycerol lipase beta – Another enzyme involved in 2-AG production, with a role in immune system regulation.
Faah	FAAH	Fatty acid amide hydrolase – Enzyme that degrades anandamide (AEA), terminating its signaling activity.
Mgll	MAGL	Monoacylglycerol lipase – Enzyme that breaks down 2-AG, regulating endocannabinoid signaling.

Gene	Protein	Full Name / Function
Iba1	IBA1	Ionized calcium-binding adapter molecule 1 – Microglial activation marker, involved in neuroinflammation.
Gfap	GFAP	Glial fibrillary acidic protein – Astrocyte marker, key for gliosis and astrocyte response to injury.
Mrc1	MRC1	Mannose receptor C-type 1 – Involved in microglial/macrophage function and endocytosis.
Fcgr2b	FcγRIIb	Fc gamma receptor IIb – Regulates immune responses, inhibits excessive inflammation.
Vimentin	VIM	Vimentin – Intermediate filament protein, involved in astrocyte cytoskeleton organization and gliosis.

Gene	Protein	Full Name / Function
Il1β	IL-1β	Interleukin-1 beta – Key pro-inflammatory cytokine, mediates immune and inflammatory responses.
Ikbkb	IKKβ	Inhibitor of nuclear factor kappa-B kinase subunit beta – Regulates NF-κB signaling, critical for inflammation.
Nos2	iNOS	Inducible nitric oxide synthase – Produces nitric oxide (NO), involved in immune defense and inflammation.
Ptgs2	COX-2	Prostaglandin-endoperoxide synthase 2 (Cyclooxygenase-2) – Enzyme involved in prostaglandin synthesis and inflammation.
Nfkb1	NF-κB1 (p50)	NF-kappa B p50 subunit – The main activator of the classical NF-κB pathway, regulates inflammatory gene expression.
Rela	RelA (p65)	NF-κB p65 subunit – Key transcription factor regulating immune and inflammatory responses.

Table S2. Primer references for TaqMan® Gene Expression Assays

Genes	Accession no	Assay ID	Amplicon length
Cnr1	NM_012784.4	Rn02758689_s1	92
Cnr2	NM_020543.4	Rn01637601_s1	68
Pparα	NM_013196.1	Rn00566193_m1	98
Daglα	NM_001005886.1	Rn01454303_m1	61
Daglβ	NM_001107120.1	Rn01453770_m1	57
Mgll	NM_138502.2	Rn00593297_m1	78
Nape-pld	NM_199381.1	Rn01786262_m1	71
Faah	NM_024132.3	Rn00577086 _m1	63
Iba1 (Aif1)	NM_017196.3	Rn00574125_g1	126
Gfap	NM_017009.2	Rn01253033_m1	75
Mrc1	NM_001106123.2	Rn01487342_m1	62
Fcgr2b	NM_175756.1	Rn01490232_m1	60
Il1b	NM_031512.2	Rn00580432_m1	74
Ikbkb (IKKβ)	NM_053355.2	Rn00584379_m1	84
Nos2	NM_012611.3	Rn00561646_m1	77
Ptgs2 (COX2)	NM_017232.3	Rn01483828_m1	112
Rela (NF-κB)	NM_199267.2	Rn01502266_m1	67
Actb	NM_031144.2	Rn00667869_m1	91

Abbreviations: *Actb*, actin beta; *Cnr1*, cannabinoid receptor 1 (brain); *Cnr2*, cannabinoid receptor 2 (macrophage); *Daglα*, diacylglycerol lipase, alpha; *Daglβ*, diacylglycerol lipase, beta; *Faah*, fatty acid amide hydrolase, *Fcgr2b*, Fc fragment of IgG, low affinity IIb, receptor; *Gfap*, glial fibrillary acidic protein; *Iba1*, ionized calcim-binding adapter molecule 1 (allograft inflammatory factor 1); *Ikbkb*, inhibitor of kappa light polypeptide gene enhancer in B-cells, kinase beta; *Il1b*, interleukin 1 beta; *Mgll*, monoglyceride lipase; *Mrc1*, mannose receptor, C type 1; *Nape-pld*, N-acyl phosphatidylethanolamine phospholipase D; *Nos2*, nitric oxide synthase 2, inducible; *Pparα*, peroxisome proliferator activated receptor alpha; *Ptgs2*, prostaglandin-endoperoxide synthase 2 (cyclooxygenase-2); *Rela*, v-rel avian reticuloendotheliosis viral oncogene homolog A (nuclear factor kappa B).

Table S3. Primary antibodies used

Antigen	Immunogen	Manufacturing details	Dilution	Predicted molecular weight (kDa)
CB1	Synthetic peptide: MSVSTDTSAE AL, corresponding to C terminal amino acid 461-472 of Human Cannabinoid Receptor I.	Abcam<br>Polyclonal IgG antibody<br>Produced in rabbit<br>Code No.: ab23703<br>RRID:AB_447623	1:200	53
CB2	Fusion proteinm corresponding to amino acids 1-32 of Rat Cannabinoid Receptor II.	Abcam<br>Polyclonal IgG antibody<br>Produced in rabbit<br>Code No.: ab3561	1:200	40
PPARα	Synthetic peptide corresponding to residues M(1)VDTESPICPLSPLEADD(18)C of mouse PPAR alpha	Fitzgerald<br>Polyclonal IgG antibody<br>Produced in rabbit<br>Code No.: 20R-PR021<br>Lot. No.: P15092226	1:200	52
DAGLα	KLH conjugated synthetic peptide derived from human DAGLA	BioNova<br>Polyclonal IgG antibody<br>Produced in rabbit<br>Code No.: orb156533	1:100	115
DAGLβ	KLH conjugated synthetic peptide derived from human DAGLB	BioNova<br>Polyclonal IgG antibody<br>Produced in rabbit<br>Code No.: orb182976	1:100	74
MAGL	Human monoacylglycerol lipase amino acids 1-14: MPEESSPRRTPQSI	Cayman Chemical<br>Polyclonal IgG antibody<br>Produced in rabbit<br>Code No.: 100035<br>Lot. No.: 163084	1:200	35
NAPE-PLD	Human NAPE-PLD amino acids 6-20: SNQSLMTSSQYPKEA	Cayman Chemical<br>Polyclonal IgG antibody<br>Produced in rabbit<br>Code No.: 10306	1:100	46
FAAH	Synthetic peptide from rat fatty acid amid hydrolase amino acids 561-579 (CLRFMREVEQLMTPQKQPS) conjugated to KLK	Cayman Chemical<br>Polyclonal IgG antibody<br>Produced in rabbit<br>Code No.: 101600<br>Lot. No.: 157878	1:200	63
IBA1	Linear peptide corresponding to human Iba1/AIF1	Millipore<br>Monoclonal IgG antibody<br>Produced in mouse<br>Code No.: MABN92<br>Lot. No.: 2383608	1:500	17
GFAP	Purified GFAP from pig spinal cord	Sigma<br>Monoclonal IgG antibody<br>Produced in mouse<br>Code No.: G3893	1:500	48

VIM	Human thymic nuclear extract	Sigma<br>Monoclonal IgG antibody<br>Produced in mouse<br>Code No.: V6630	1:200	58
IKKβ	Synthetic peptide corresponding to residues near the carboxy terminus of human IKKβ protein	CellSignaling<br>Monoclonal IgG antibody<br>Produced in rabbit<br>Code No.: 8943	1:1000	87
COX2	Synthetic peptide corresponding to residues surrounding His108 of human Cox2 protein	CellSignaling<br>Monoclonal IgG antibody<br>Produced in rabbit<br>Code No.: 122821	1:500	74
NF-κB	Synthetic peptide corresponding to residues surrounding Glu498 of human NF-κB p65/RelA protein	CellSignaling<br>Monoclonal IgG antibody<br>Produced in rabbit<br>Code No.: 8242	1:1000	65
γ-Adaptin	Mouse adaptin γ amino acids 642-821	BD Biosciences<br>Monoclonal IgG<br>Produced in mouse<br>Code No.: 610385	1:2000	104

Abbreviations: VIM, vimentin

Table S4. Two-way ANOVA showing interaction and main effects of dependence and drug on the mRNA expression of main components of the endocannabiboid system in the prefrontal cortex.

	Source of Variation	F (DFn, DFd)	P value
Cnr1	Interaction	F (2, 42) = 0.8843	ns
	Dependence	F (1, 42) = 4.239	***0.0457***
	Drug	F (2, 42) = 1.352	ns
Cnr2	Interaction	F (2, 42) = 6.722	***0.0029***
	Dependence	F (1, 42) = 12.95	***0.0008***
	Drug	F (2, 42) = 2.284	ns
Ppara	Interaction	F (2, 42) = 0.5126	ns
	Dependence	F (1, 42) = 30.59	***<0.0001***
	Drug	F (2, 42) = 3.923	***0.0274***
Dagla	Interaction	F (2, 42) = 2.842	***0.0695***
	Dependence	F (1, 42) = 0.4525	ns
	Drug	F (2, 42) = 0.6112	ns
Daglb	Interaction	F (2, 42) = 4.076	***0.0241***
	Dependence	F (1, 42) = 9.870	***0.0031***
	Drug	F (2, 42) = 0.4598	ns
Mgll	Interaction	F (2, 42) = 2.896	***0.0663***
	Dependence	F (1, 42) = 0.9844	ns
	Drug	F (2, 42) = 0.5993	ns
Napepld	Interaction	F (2, 42) = 1.811	ns
	Dependence	F (1, 42) = 0.8492	ns
	Drug	F (2, 42) = 0.1386	ns
Faah	Interaction	F (2, 42) = 4.234	***0.0211***
	Dependence	F (1, 42) = 8.965	***0.0046***
	Drug	F (2, 42) = 1.121	ns

ns, not significant

Table S5. Two-way ANOVA showing interaction and main effects of dependence and drug on the protein levels of main components of the endocannabiboid system in the prefrontal cortex.

	Source of Variation	F (DFn, DFd)	P value
CB1	Interaction	F (2, 19) = 0.331	ns
	Dependence	F (1, 19) = 7.750	***0.0118***
	Drug	F (2, 19) = 11.45	***0.0005***
CB2	Interaction	F (2, 19) = 1.812	ns
	Dependence	F (1, 19) = 6.828	***0.0171***
	Drug	F (2, 19) = 6.496	***0.0071***
PPARα	Interaction	F (2, 19) = 12.15	***0.0004***
	Dependence	F (1, 19) = 0.038	ns
	Drug	F (2, 19) = 4.183	***0.0312***
DAGLα	Interaction	F (2, 19) = 2.829	ns
	Dependence	F (1, 19) = 27.19	***<0.0001***
	Drug	F (2, 19) = 3.681	***0.0445***
DAGLβ	Interaction	F (2, 19) = 7.568	***0.0038***
	Dependence	F (1, 19) = 1.653	ns
	Drug	F (2, 19) = 12.44	***0.0004***
MAGL	Interaction	F (2, 19) = 5.977	***0.0097***
	Dependence	F (1, 19) = 88.41	***<0.0001***
	Drug	F (2, 19) = 29.48	***<0.0001***
NAPE-PLD	Interaction	F (2, 19) = 12.37	***0.0004***
	Dependence	F (1, 19) = 0.807	ns
	Drug	F (2, 19) = 2.955	ns
FAAH	Interaction	F (2, 19) = 44.71	***<0.0001***
	Dependence	F (1, 19) = 38.53	***<0.0001***
	Drug	F (2, 19) = 3.940	***0.037***

ns, not significant

Table S6. Two-way ANOVA showing interaction and main effects of dependence and drug on the mRNA expression of main components of gliosis and inflammation in the prefrontal cortex.

	Source of Variation	F (DFn, DFd)	P value
Iba1	Interaction	F (2, 42) = 0.591	ns
	Dependence	F (1, 42) = 1.427	ns
	Drug	F (2, 42) = 0.056	ns
Gfap	Interaction	F (2, 42) = 3.014	***0.0598***
	Dependence	F (1, 42) = 0.295	ns
	Drug	F (2, 42) = 1.284	ns
Mrc1	Interaction	F (2, 42) = 0.647	ns
	Dependence	F (1, 42) = 12.58	***0.001***
	Drug	F (2, 42) = 0.766	ns
Fcgr2b	Interaction	F (2, 42) = 0.944	ns
	Dependence	F (1, 42) = 0.937	ns
	Drug	F (2, 42) = 4.159	***0.0225***
Il1b	Interaction	F (2, 42) = 0.738	ns
	Dependence	F (1, 42) = 6.953	***0.0117***
	Drug	F (2, 42) = 1.341	ns
Ikbkb	Interaction	F (2, 42) = 3.520	***0.0386***
	Dependence	F (1, 42) = 8.359	***0.0061***
	Drug	F (2, 42) = 0.012	ns
Nos2	Interaction	F (2, 42) = 2.319	ns
	Dependence	F (1, 42) = 9.709	***0.0033***
	Drug	F (2, 42) = 0.320	ns
Ptgs2	Interaction	F (2, 42) = 2.800	ns
	Dependence	F (1, 42) = 13.83	***0.0006***
	Drug	F (2, 42) = 3.653	***0.0344***
Rela	Interaction	F (2, 42) = 3.501	***0.0392***
	Dependence	F (1, 42) = 5.645	***0.0221***
	Drug	F (2, 42) = 5.187	***0.0097***

ns, not significant

Table S7. Two-way ANOVA showing interaction and main effects of dependence and drug on the protein levels of main components of gliosis and inflammation in the prefrontal cortex.

	Source of Variation	F (DFn, DFd)	P value
IBA1	Interaction	F (2, 19) = 13.32	***0.0002***
	Dependence	F (1, 19) = 34.40	***<0.0001***
	Drug	F (2, 19) = 0.883	ns
GFAP	Interaction	F (2, 19) = 6.839	***0.0058***
	Dependence	F (1, 19) = 2.779	ns
	Drug	F (2, 19) = 3.212	***0.0628***
Vimentin	Interaction	F (2, 19) = 2.544	ns
	Dependence	F (1, 19) = 0.042	ns
	Drug	F (2, 19) = 20.78	***<0.0001***
COX2	Interaction	F (2, 19) = 0.625	ns
	Dependence	F (1, 19) = 0.0003	ns
	Drug	F (2, 19) = 2.431	ns
NF-κB	Interaction	F (2, 19) = 6.111	***0.0089***
	Dependence	F (1, 19) = 4.425	***0.049***
	Drug	F (2, 19) = 8.371	***0.0025***
IKKβ	Interaction	F (2, 19) = 17.87	***<0.0001***
	Dependence	F (1, 19) = 48.89	***<0.0001***
	Drug	F (2, 19) = 39.09	***<0.0001***

ns, not significant

Table S8. Two-way ANOVA showing interaction and main effects of dependence and drug on the mRNA expression of main components of the endocannabiboid system in the hippocampus.

	Source of Variation	F (DFn, DFd)	P value
Cnr1	Interaction	F (2, 42) = 0.148	ns
	Dependence	F (1, 42) = 1.739	ns
	Drug	F (2, 42) = 2.338	ns
Cnr2	Interaction	F (2, 42) = 12.25	***<0.0001***
	Dependence	F (1, 42) = 0.648	ns
	Drug	F (2, 42) = 3.481	***0.0399***
Ppara	Interaction	F (2, 42) = 2.449	ns
	Dependence	F (1, 42) = 12.20	***0.0011***
	Drug	F (2, 42) = 3.701	***0.0331***
Dagla	Interaction	F (2, 42) = 2.161	ns
	Dependence	F (1, 42) = 17.49	***0.0001***
	Drug	F (2, 42) = 2.620	ns
Daglb	Interaction	F (2, 42) = 4.609	***0.0155***
	Dependence	F (1, 42) = 2.889	ns
	Drug	F (2, 42) = 0.153	ns
Mgll	Interaction	F (2, 42) = 6.569	***0.0033***
	Dependence	F (1, 42) = 103.2	***<0.0001***
	Drug	F (2, 42) = 0.147	ns
Napepld	Interaction	F (2, 42) = 0.906	ns
	Dependence	F (1, 42) = 0.674	ns
	Drug	F (2, 42) = 1.688	ns
Faah	Interaction	F (2, 42) = 12.85	***<0.0001***
	Dependence	F (1, 42) = 1.203	ns
	Drug	F (2, 42) = 0.475	ns

ns, not significant

Table S9. Two-way ANOVA showing interaction and main effects of dependence and drug on the protein levels of main components of the endocannabiboid system in the hippocampus.

	Source of Variation	F (DFn, DFd)	P value
CB1	Interaction	F (2, 19) = 10.70	***0.0008***
	Dependence	F (1, 19) = 0.372	ns
	Drug	F (2, 19) = 2.667	ns
CB2	Interaction	F (2, 19) = 0.545	ns
	Dependence	F (1, 19) = 10.51	***0.0043***
	Drug	F (2, 19) = 0.030	ns
PPARα	Interaction	F (2, 19) = 1.926	ns
	Dependence	F (1, 19) = 8.540	***0.0087***
	Drug	F (2, 19) = 0.662	ns
DAGLα	Interaction	F (2, 19) = 2.860	ns
	Dependence	F (1, 19) = 2.388	ns
	Drug	F (2, 19) = 18.32	***<0.0001***
DAGLβ	Interaction	F (2, 19) = 3.168	***0.065***
	Dependence	F (1, 19) = 1.020	ns
	Drug	F (2, 19) = 4.578	***0.0238***
MAGL	Interaction	F (2, 19) = 1.040	ns
	Dependence	F (1, 19) = 4.128	***0.0564***
	Drug	F (2, 19) = 1.005	ns
NAPE-PLD	Interaction	F (2, 19) = 1.250	ns
	Dependence	F (1, 19) = 3.070	ns
	Drug	F (2, 19) = 1.818	ns
FAAH	Interaction	F (2, 19) = 1.809	ns
	Dependence	F (1, 19) = 0.167	ns
	Drug	F (2, 19) = 2.164	ns

ns, not significant

Table S10. Two-way ANOVA showing interaction and main effects of dependence and drug on the mRNA expression of main components of gliosis and inflammation in the hippocampus.

	Source of Variation	F (DFn, DFd)	P value
	Interaction	F (2, 42) = 1.358	ns
Iba1	Dependence	F (1, 42) = 17.72	***0.0001***
	Drug	F (2, 42) = 1.721	ns
	Interaction	F (2, 42) = 4.172	***0.0222***
Gfap	Dependence	F (1, 42) = 20.28	***<0.0001***
	Drug	F (2, 42) = 1.886	ns
	Interaction	F (2, 42) = 0.842	ns
Mrc1	Dependence	F (1, 42) = 12.41	***0.001***
	Drug	F (2, 42) = 5.032	***0.011***
	Interaction	F (2, 42) = 5.496	***0.0076***
Fcgr2b	Dependence	F (1, 42) = 0.453	ns
	Drug	F (2, 42) = 3.621	***0.0354***
	Interaction	F (2, 42) = 0.360	ns
Il1b	Dependence	F (1, 42) = 17.10	***0.0002***
	Drug	F (2, 42) = 1.891	ns
	Interaction	F (2, 42) = 3.770	***0.0312***
Ikbkb	Dependence	F (1, 42) = 148.6	***<0.0001***
	Drug	F (2, 42) = 0.473	ns
	Interaction	F (2, 42) = 8.321	***0.0009***
Nos2	Dependence	F (1, 42) = 9.949	***0.003***
	Drug	F (2, 42) = 3.769	***0.0312***
	Interaction	F (2, 42) = 0.836	ns
Ptgs2	Dependence	F (1, 42) = 25.65	***<0.0001***
	Drug	F (2, 42) = 0.063	ns
	Interaction	F (2, 42) = 3.150	***0.0531***
Rela	Dependence	F (1, 42) = 20.46	***<0.0001***
	Drug	F (2, 42) = 19.88	***<0.0001***

ns, not significant

Table S11. Two-way ANOVA showing interaction and main effects of dependence and drug on the protein levels of main components of gliosis and inflammation in the hippocampus.

	Source of Variation	F (DFn, DFd)	P value
IBA1	Interaction	F (2, 19) = 2.639	ns
	Dependence	F (1, 19) = 22.31	***0.0001***
	Drug	F (2, 19) = 6.472	***0.0072***
GFAP	Interaction	F (2, 19) = 1.211	ns
	Dependence	F (1, 19) = 6.005	***0.0241***
	Drug	F (2, 19) = 2.031	ns
Vimentin	Interaction	F (2, 19) = 2.544	ns
	Dependence	F (1, 19) = 0.042	ns
	Drug	F (2, 19) = 20.78	***<0.0001***
COX2	Interaction	F (2, 19) = 1.131	ns
	Dependence	F (1, 19) = 5.926	***0.0249***
	Drug	F (2, 19) = 7.690	***0.0036***
NF-κB	Interaction	F (2, 19) = 3.595	***0.0474***
	Dependence	F (1, 19) = 0.164	ns
	Drug	F (2, 19) = 0.603	ns
IKKβ	Interaction	F (2, 19) = 1.064	ns
	Dependence	F (1, 19) = 6.136	***0.0228***
	Drug	F (2, 19) = 4.498	***0.0252***

ns, not significant

Table S12. Corresponding p values for Spearman correlation of genes corresponding to the endocannabinoid system, gliosis and inflammation during repeated administration of WIN 55,212-2 and HU-210 in the prefrontal cortex.

	Cnr1	*Cnr2*	*Ppara*	*Dagla*	*Daglb*	*Mgll*	*Napepld*	*Faah*	*Iba1*	*Gfap*	*Mrc1*	*Fcgr2b*	*Il1b*	*Ikbkb*	*Nos2*	*Ptgs2*	*Rela*
Cnr1		0.496	0.125	0.031	0.029	0.033	0.002	0.011	0.237	0.011	0.525	0.953	0.360	0.035	0.048	0.412	0.060
Cnr2	0.496		0.548	0.498	0.134	0.942	0.673	0.351	0.304	0.820	0.304	0.809	0.402	0.851	0.787	0.204	0.074
Ppara	0.125	0.548		0.475	0.347	0.174	0.070	0.360	0.239	0.000	0.037	0.581	0.090	0.014	0.375	0.450	0.576
Dagla	0.031	0.498	0.475		0.013	0.000	0.185	0.000	0.082	0.465	0.974	0.682	0.974	0.000	0.000	0.004	0.048
Daglb	0.029	0.134	0.347	0.013		0.044	0.262	0.043	0.527	0.146	0.713	0.605	0.965	0.008	0.015	0.560	0.000
Mgll	0.033	0.942	0.174	0.000	0.044		0.281	0.000	0.131	0.240	0.310	0.689	0.453	0.000	0.001	0.019	0.043
Napepld	0.002	0.673	0.070	0.185	0.262	0.281		0.197	0.539	0.004	0.106	0.709	0.657	0.041	0.007	0.872	0.904
Faah	0.011	0.351	0.360	0.000	0.043	0.000	0.197		0.063	0.424	0.815	0.369	0.793	0.003	0.000	0.001	0.092
Iba1	0.237	0.304	0.239	0.082	0.527	0.131	0.539	0.063		0.275	0.549	0.173	0.370	0.200	0.280	0.225	0.815
Gfap	0.011	0.820	0.000	0.465	0.146	0.240	0.004	0.424	0.275		0.026	0.910	0.557	0.027	0.204	0.668	0.379
Mrc1	0.525	0.304	0.037	0.974	0.713	0.310	0.106	0.815	0.549	0.026		0.971	0.018	0.787	0.878	0.768	0.265
Fcgr2b	0.953	0.809	0.581	0.682	0.605	0.689	0.709	0.369	0.173	0.910	0.971		0.916	0.788	0.192	0.835	0.868
Il1b	0.360	0.402	0.090	0.974	0.965	0.453	0.657	0.793	0.370	0.557	0.018	0.916		0.974	0.686	0.268	0.971
Ikbkb	0.035	0.851	0.014	0.000	0.008	0.000	0.041	0.003	0.200	0.027	0.787	0.788	0.974		0.000	0.187	0.026
Nos2	0.048	0.787	0.375	0.000	0.015	0.001	0.007	0.000	0.280	0.204	0.878	0.192	0.686	0.000		0.062	0.049
Ptgs2	0.412	0.204	0.450	0.004	0.560	0.019	0.872	0.001	0.225	0.668	0.768	0.835	0.268	0.187	0.062		0.722
Rela	0.060	0.074	0.576	0.048	0.000	0.043	0.904	0.092	0.815	0.379	0.265	0.868	0.971	0.026	0.049	0.722	

Table S13. Corresponding p values for Spearman correlation of genes corresponding to the endocannabinoid system, gliosis and inflammation during withdrawal of WIN 55,212-2 and HU-210 in the prefrontal cortex.

	Cnr1	*Cnr2*	*Ppara*	*Dagla*	*Daglb*	*Mgll*	*Napepld*	*Faah*	*Iba1*	*Gfap*	*Mrc1*	*Fcgr2b*	*Il1b*	*Ikbkb*	*Nos2*	*Ptgs2*	*Rela*
Cnr1		0.952	0.862	0.572	0.064	0.509	0.277	0.342	0.448	0.683	0.728	0.939	0.300	0.252	0.501	0.027	0.043
Cnr2	0.952		0.832	0.795	0.126	0.353	0.492	0.254	0.024	0.245	0.569	0.003	0.576	0.709	0.543	0.410	0.161
Ppara	0.862	0.832		0.354	0.395	0.493	0.276	0.405	0.306	0.306	0.105	0.284	0.549	0.762	0.692	0.625	0.248
Dagla	0.572	0.795	0.354		0.006	0.000	0.002	0.002	0.864	0.017	0.931	0.609	0.091	0.065	0.712	0.001	0.835
Daglb	0.064	0.126	0.395	0.006		0.001	0.019	0.033	0.246	0.182	0.421	0.259	0.028	0.173	0.036	0.005	0.107
Mgll	0.509	0.353	0.493	0.000	0.001		0.006	0.000	0.965	0.063	0.747	0.093	0.064	0.013	0.113	0.000	0.778
Napepld	0.277	0.492	0.276	0.002	0.019	0.006		0.221	0.554	0.001	0.630	0.742	0.211	0.973	0.629	0.115	0.380
Faah	0.342	0.254	0.405	0.002	0.033	0.000	0.221		0.853	0.990	0.336	0.203	0.001	0.008	0.142	0.002	0.083
Iba1	0.448	0.024	0.306	0.864	0.246	0.965	0.554	0.853		0.334	0.010	0.017	0.007	0.220	0.113	0.802	0.051
Gfap	0.683	0.245	0.306	0.017	0.182	0.063	0.001	0.990	0.334		0.071	0.045	0.316	0.288	0.286	0.171	0.046
Mrc1	0.728	0.569	0.105	0.931	0.421	0.747	0.630	0.336	0.010	0.071		0.046	0.001	0.022	0.028	0.923	0.015
Fcgr2b	0.939	0.003	0.284	0.609	0.259	0.093	0.742	0.203	0.017	0.045	0.046		0.139	0.223	0.125	0.223	0.117
Il1b	0.300	0.576	0.549	0.091	0.028	0.064	0.211	0.001	0.007	0.316	0.001	0.139		0.001	0.001	0.076	0.016
Ikbkb	0.252	0.709	0.762	0.065	0.173	0.013	0.973	0.008	0.220	0.288	0.022	0.223	0.001		0.001	0.007	0.096
Nos2	0.501	0.543	0.692	0.712	0.036	0.113	0.629	0.142	0.113	0.286	0.028	0.125	0.001	0.001		0.353	0.038
Ptgs2	0.027	0.410	0.625	0.001	0.005	0.000	0.115	0.002	0.802	0.171	0.923	0.223	0.076	0.007	0.353		0.571
Rela	0.043	0.161	0.248	0.835	0.107	0.778	0.380	0.083	0.051	0.046	0.015	0.117	0.016	0.096	0.038	0.571	

Table S14. Corresponding p values for Spearman correlation of genes corresponding to the endocannabinoid system, gliosis and inflammation during repeated administration of WIN 55,212-2 and HU-210 in the hippocampus.

	Cnr1	*Cnr2*	*Ppara*	*Dagla*	*Daglb*	*Mgll*	*Napepld*	*Faah*	*Iba1*	*Gfap*	*Mrc1*	*Fcgr2b*	*Il1b*	*Ikbkb*	*Nos2*	*Ptgs2*	*Rela*
Cnr1		0.179	0.001	0.004	0.012	0.756	0.005	0.005	0.003	0.012	0.000	0.000	0.660	0.009	0.491	0.001	0.668
Cnr2	0.179		0.961	0.639	0.961	0.367	0.971	0.971	0.658	0.945	0.010	0.725	0.366	0.880	0.000	0.929	0.020
Ppara	0.001	0.961		0.049	0.105	0.501	0.028	0.028	0.158	0.159	0.210	0.000	0.379	0.023	0.511	0.011	0.778
Dagla	0.004	0.639	0.049		0.005	0.271	0.001	0.001	0.027	0.001	0.035	0.045	0.098	0.001	0.853	0.007	0.660
Daglb	0.012	0.961	0.105	0.005		0.106	0.000	0.000	0.001	0.000	0.023	0.021	0.913	0.007	0.409	0.001	0.622
Mgll	0.756	0.367	0.501	0.271	0.106		0.916	0.916	0.731	0.206	0.968	0.349	0.197	0.382	0.088	0.767	0.277
Napepld	0.005	0.971	0.028	0.001	0.000	0.916		0.000	0.000	0.000	0.004	0.009	0.784	0.014	0.428	0.000	0.384
Faah	0.005	0.971	0.028	0.001	0.000	0.916	0.000		0.000	0.000	0.004	0.009	0.784	0.014	0.428	0.000	0.384
Iba1	0.003	0.658	0.158	0.027	0.001	0.731	0.000	0.000		0.000	0.012	0.000	0.308	0.011	0.878	0.000	0.409
Gfap	0.012	0.945	0.159	0.001	0.000	0.206	0.000	0.000	0.000		0.029	0.035	0.579	0.013	0.424	0.001	0.438
Mrc1	0.000	0.010	0.210	0.035	0.023	0.968	0.004	0.004	0.012	0.029		0.013	0.599	0.184	0.087	0.022	0.734
Fcgr2b	0.000	0.725	0.000	0.045	0.021	0.349	0.009	0.009	0.000	0.035	0.013		0.657	0.010	0.662	0.002	0.443
Il1b	0.660	0.366	0.379	0.098	0.913	0.197	0.784	0.784	0.308	0.579	0.599	0.657		0.175	0.177	0.875	0.961
Ikbkb	0.009	0.880	0.023	0.001	0.007	0.382	0.014	0.014	0.011	0.013	0.184	0.010	0.175		0.519	0.013	0.636
Nos2	0.491	0.000	0.511	0.853	0.409	0.088	0.428	0.428	0.878	0.424	0.087	0.662	0.177	0.519		0.554	0.004
Ptgs2	0.001	0.929	0.011	0.007	0.001	0.767	0.000	0.000	0.000	0.001	0.022	0.002	0.875	0.013	0.554		0.369
Rela	0.668	0.020	0.778	0.660	0.622	0.277	0.384	0.384	0.409	0.438	0.734	0.443	0.961	0.636	0.004	0.369	

Table S15. Corresponding p values for Spearman correlation of genes corresponding to the endocannabinoid system, gliosis and inflammation during withdrawal of WIN 55,212-2 and HU-210 in the hippocampus.

	Cnr1	*Cnr2*	*Ppara*	*Dagla*	*Daglb*	*Mgll*	*Napepld*	*Faah*	*Iba1*	*Gfap*	*Mrc1*	*Fcgr2b*	*Il1b*	*Ikbkb*	*Nos2*	*Ptgs2*	*Rela*
Cnr1		0.001	0.821	0.088	0.538	0.409	0.867	0.867	0.886	0.174	0.229	0.764	0.718	0.595	0.000	0.501	0.724
Cnr2	0.001		0.020	0.853	0.857	0.633	0.543	0.543	0.192	0.249	0.334	0.609	0.904	0.670	0.000	0.363	0.068
Ppara	0.821	0.020		0.583	0.458	0.400	0.746	0.746	0.718	0.093	0.716	0.719	0.340	0.062	0.078	0.965	0.840
Dagla	0.088	0.853	0.583		0.114	0.025	0.066	0.066	0.695	0.111	0.182	0.266	0.113	0.933	0.241	0.375	0.068
Daglb	0.538	0.857	0.458	0.114		0.072	0.300	0.300	0.095	0.212	0.024	0.084	0.132	0.067	0.628	0.625	0.394
Mgll	0.409	0.633	0.400	0.025	0.072		0.182	0.182	1.000	0.033	0.236	0.248	0.042	0.045	0.192	0.557	0.104
Napepld	0.867	0.543	0.746	0.066	0.300	0.182		0.000	0.148	0.136	0.040	0.010	0.002	0.459	0.969	0.212	0.010
Faah	0.867	0.543	0.746	0.066	0.300	0.182	0.000		0.148	0.136	0.040	0.010	0.002	0.459	0.969	0.212	0.010
Iba1	0.886	0.192	0.718	0.695	0.095	1.000	0.148	0.148		0.025	0.120	0.001	0.648	0.660	0.501	0.159	0.932
Gfap	0.174	0.249	0.093	0.111	0.212	0.033	0.136	0.136	0.025		0.010	0.001	0.062	0.271	0.103	0.045	0.030
Mrc1	0.229	0.334	0.716	0.182	0.024	0.236	0.040	0.040	0.120	0.010		0.000	0.094	0.707	0.850	0.480	0.065
Fcgr2b	0.764	0.609	0.719	0.266	0.084	0.248	0.010	0.010	0.001	0.001	0.000		0.243	0.336	0.395	0.063	0.061
Il1b	0.718	0.904	0.340	0.113	0.132	0.042	0.002	0.002	0.648	0.062	0.094	0.243		0.038	0.465	0.450	0.010
Ikbkb	0.595	0.670	0.062	0.933	0.067	0.045	0.459	0.459	0.660	0.271	0.707	0.336	0.038		0.799	0.579	0.034
Nos2	0.000	0.000	0.078	0.241	0.628	0.192	0.969	0.969	0.501	0.103	0.850	0.395	0.465	0.799		0.316	0.367
Ptgs2	0.501	0.363	0.965	0.375	0.625	0.557	0.212	0.212	0.159	0.045	0.480	0.063	0.450	0.579	0.316		0.642
Rela	0.724	0.068	0.840	0.068	0.394	0.104	0.010	0.010	0.932	0.030	0.065	0.061	0.010	0.034	0.367	0.642	

Supplementary Figure S1

Western Blots
Gels and immunoblots
Prefrontal Cortex

…involving the prefrontal cortex of Wistar rats treated with WIN 55,212-2, HU-210, and a vehicle under conditions of repeated administration and withdrawal.
Each lane represents a sample from one rat, with the following groups:

- **WIN 55,212-2 Repeated administration**: n = 4
- **HU-210 Repeated administration**: n = 4
- **Vehicle Repeated administration**: n = 5
- **WIN 55,212-2 Withdrawal**: n = 4
- **HU-210 Withdrawal**: n = 4
- **Vehicle Withdrawal**: n = 4

Gel 1: Ponceau S Red Staining

Prefrontal cortex
Main components (receptors and enzymes) of the endocannabinoid system: CB1

Group	WIN-REP				HU-REP				VEH-REP					WIN-WD				HU-WD				VEH-WD				MW (kDa)
Rat number	1	2	3	4	9	10	11	12	16	17	18	20	21	23	24	25	26	32	33	34	35	41	42	43	44	

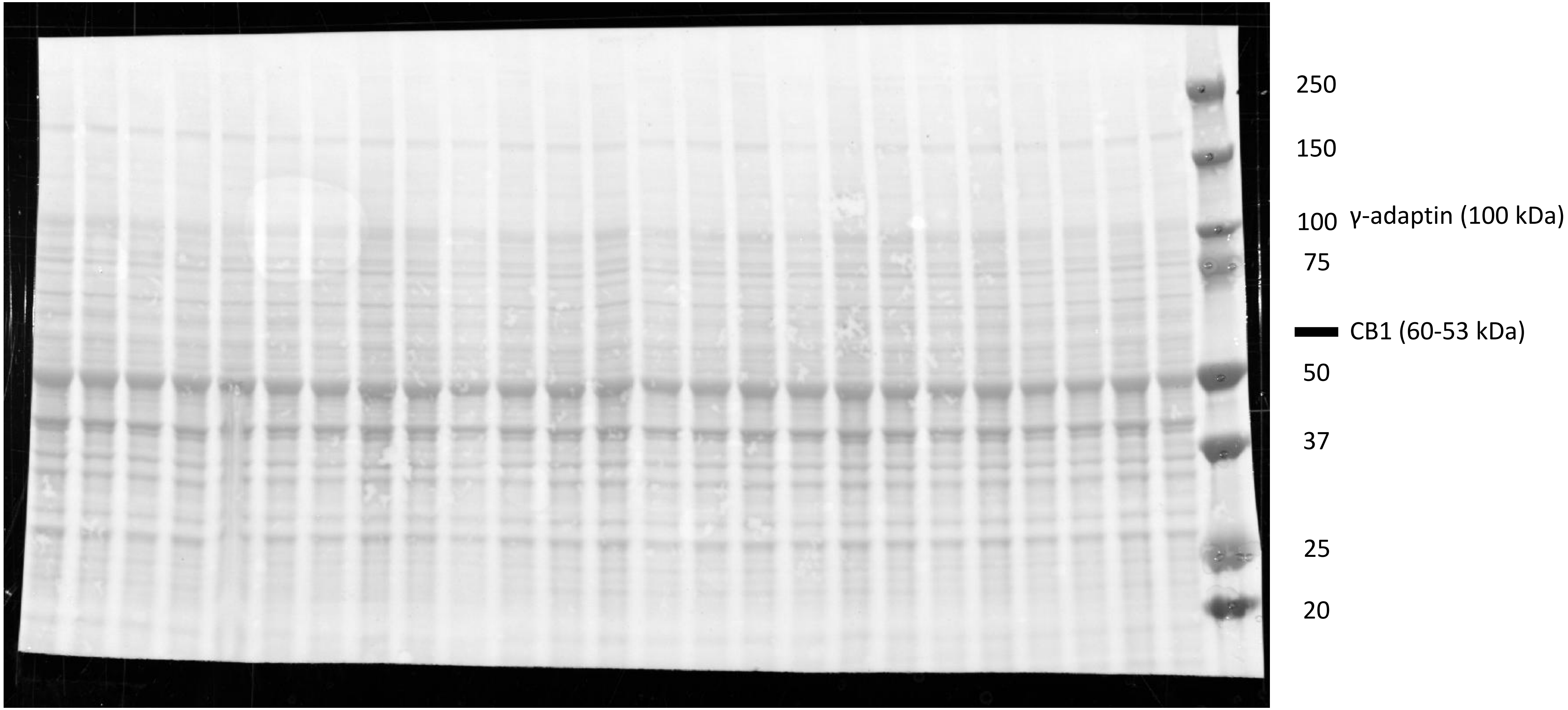

Membrane 1: Immunoblot

Prefrontal cortex
CB1 (≈ 53-60 kDa)

Group	WIN-REP				HU-REP				VEH-REP					WIN-WD				HU-WD				VEH-WD			
Rat number	1	2	3	4	9	10	11	12	16	17	18	20	21	23	24	25	26	32	33	34	35	41	42	43	44

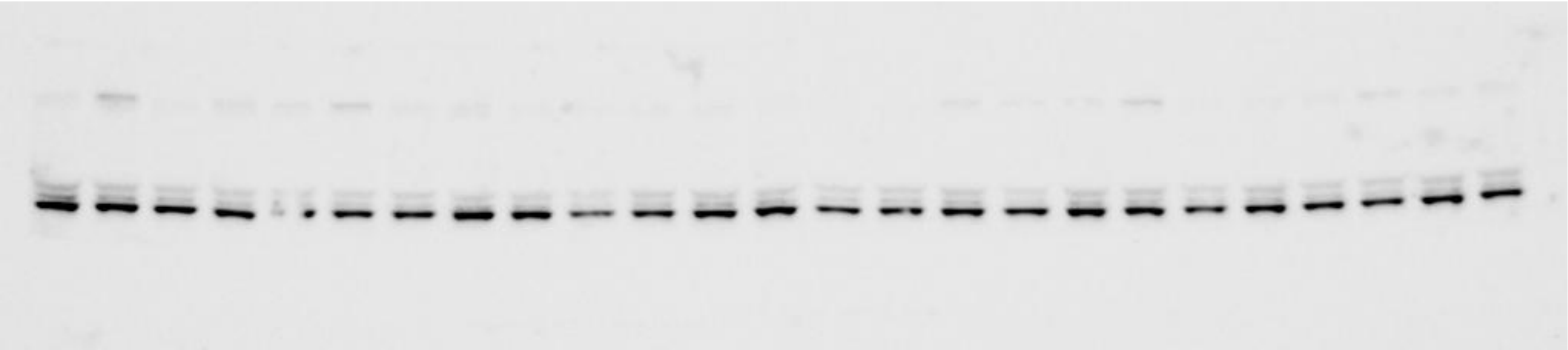

CB1

Membrane 1: Immunoblot

Seeman Prefrontal cortex
CB1 (≈ 53-60 kDa)

Group	WIN-REP				HU-REP				VEH-REP					WIN-WD				HU-WD				VEH-WD			
Rat number	1	2	3	4	9	10	11	12	16	17	18	20	21	23	24	25	26	32	33	34	35	41	42	43	44

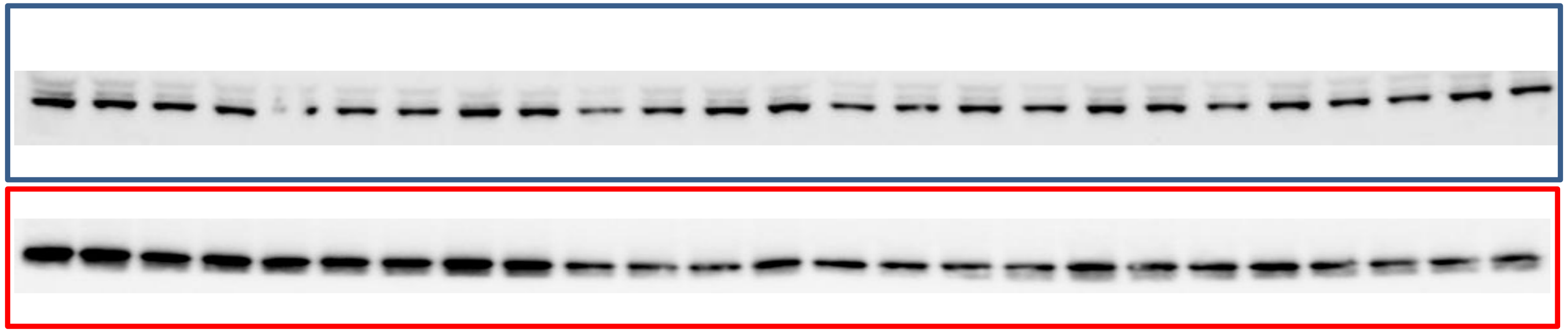

Gel 2: Ponceau S Red Staining

Prefrontal cortex

Main components (receptors and enzymes) of the endocannabinoid system: CB2

Group	MW (kDa)	WIN-REP				HU-REP				VEH-REP					WIN-WD				HU-WD				VEH-WD			
Rat number		1	2	3	4	9	10	11	12	16	17	18	20	21	23	24	25	26	32	33	34	35	41	42	43	44

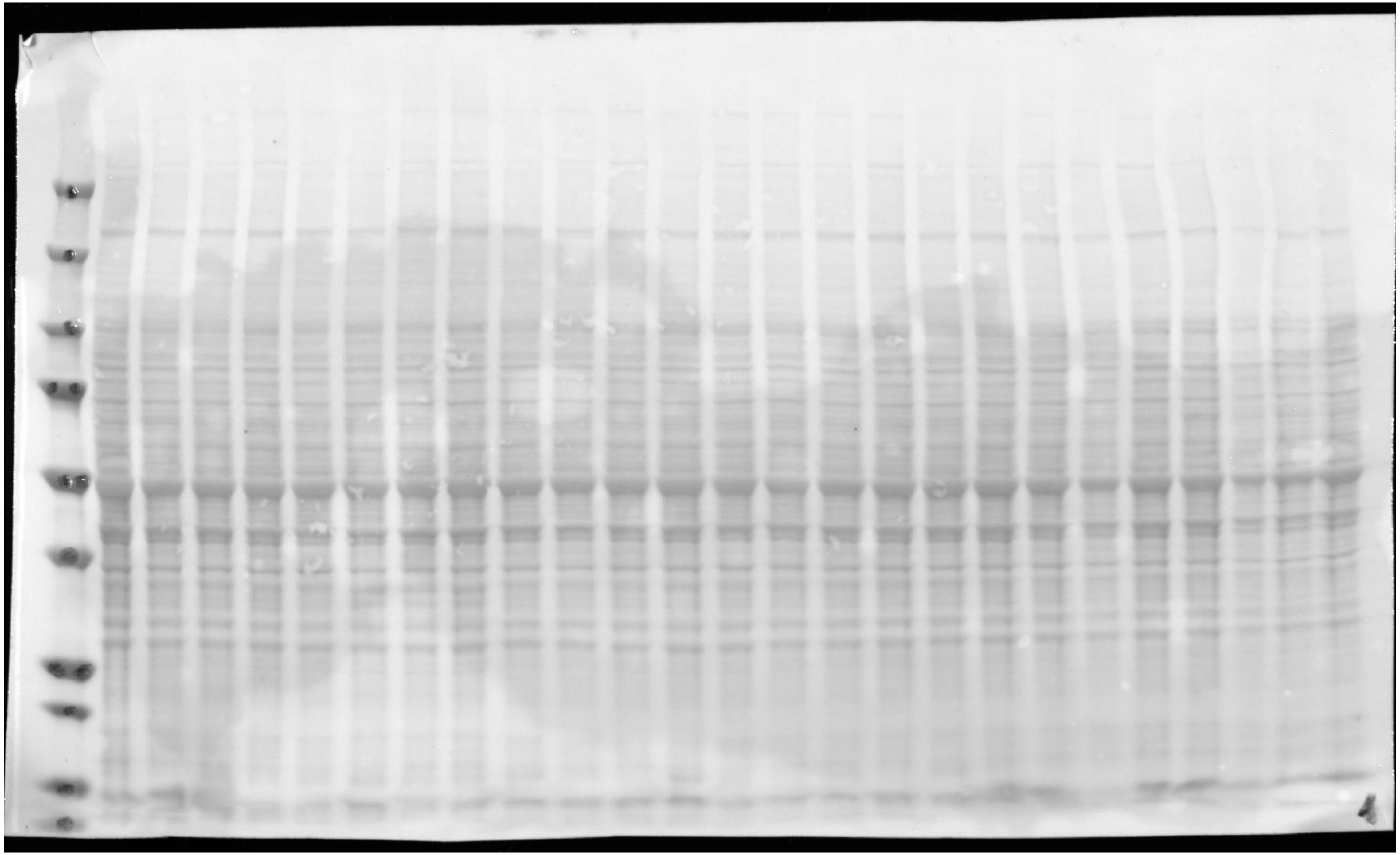

Membrane 2: Immunoblot

Prefrontal cortex
CB2 (≈ 40 kDa)

Group	WIN-REP				HU-REP				VEH-REP					WIN-WD				HU-WD				VEH-WD			
Rat number	1	2	3	4	9	10	11	12	16	17	18	20	21	23	24	25	26	32	33	34	35	41	42	43	44

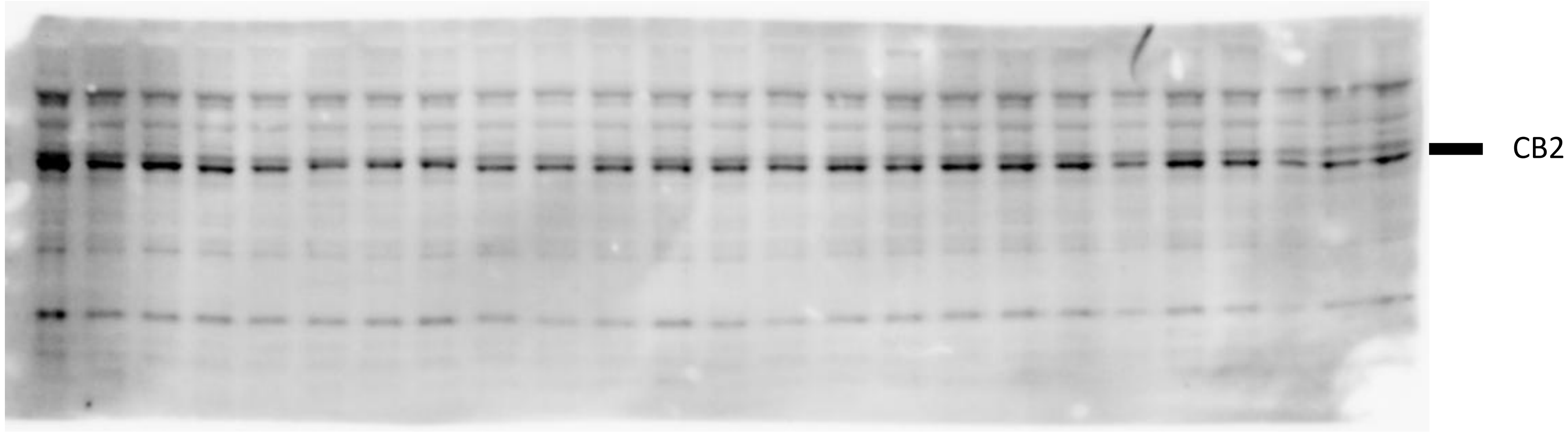

CB2

Membrane 2: Immunoblot

Prefrontal cortex
CB2 (≈ 40 kDa)

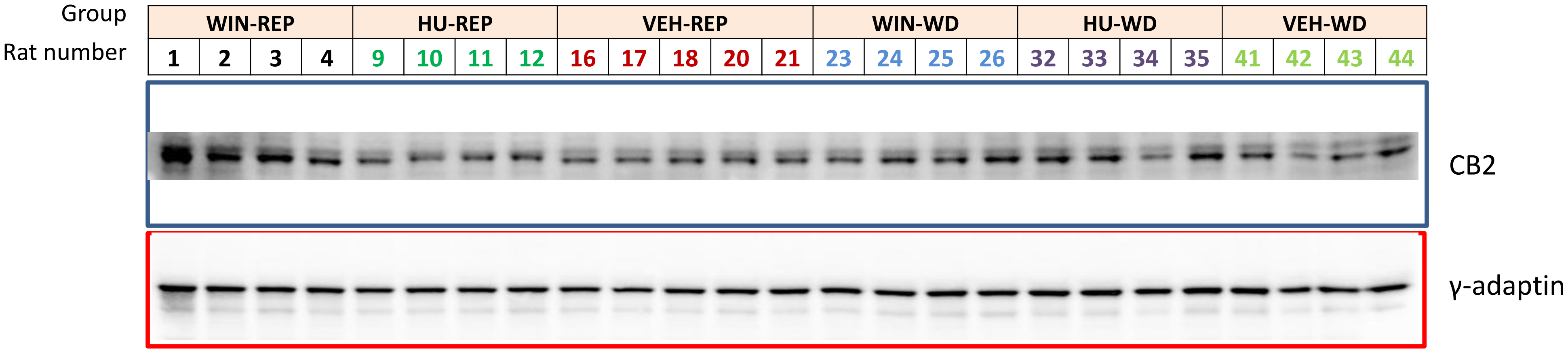

Gel 3: Ponceau S Red Staining

Prefrontal cortex

Main components (receptors and enzymes) of the endocannabinoid system: DAGLα, PPARα, FAAH

Group	WIN-REP				HU-REP				VEH-REP					WIN-WD				HU-WD				VEH-WD				MW (kDa)
Rat number	1	2	3	4	9	10	11	12	16	17	18	20	21	23	24	25	26	32	33	34	35	41	42	43	44	

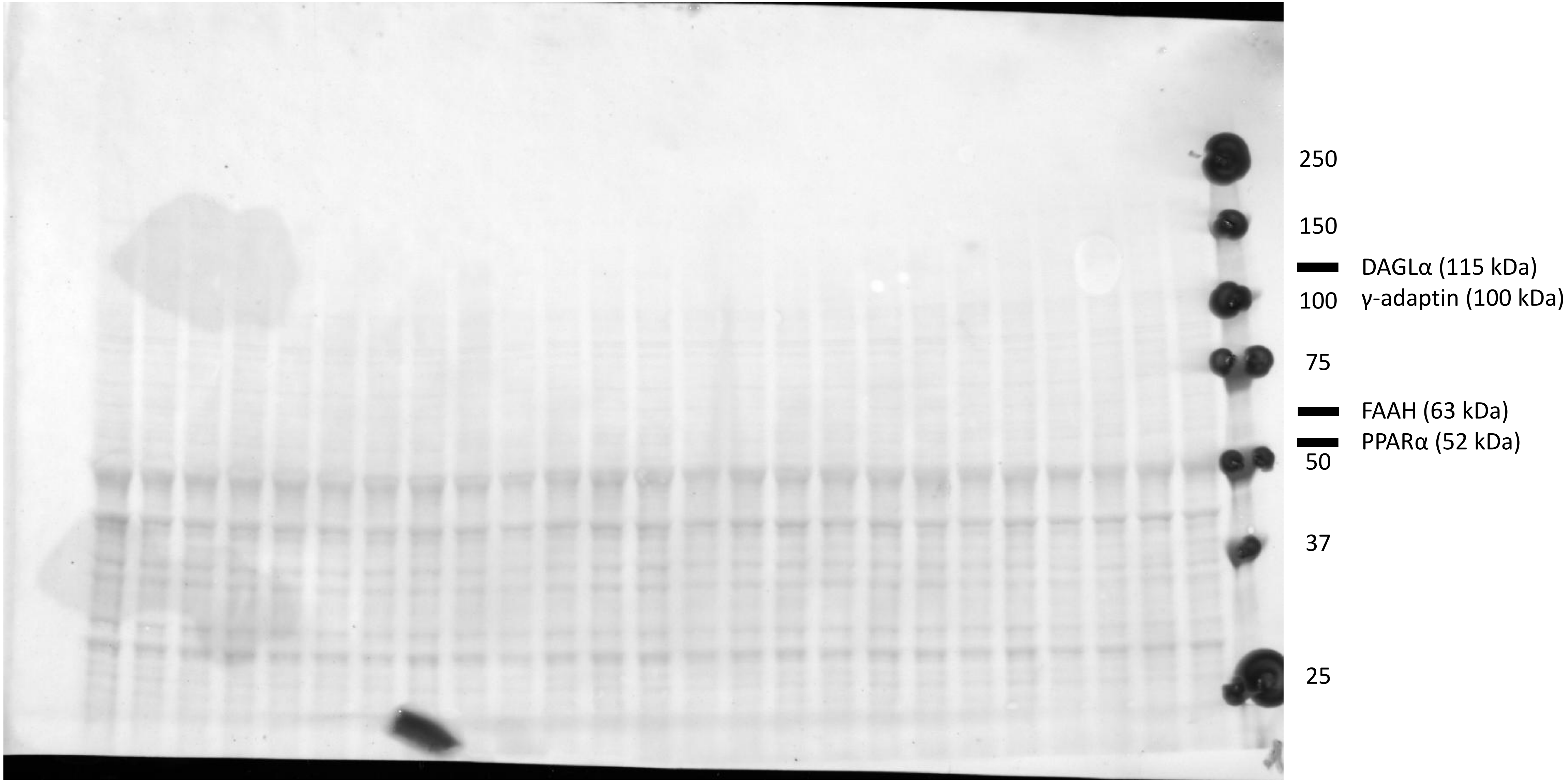

Membrane 3: Immunoblot

Prefrontal cortex
PPARα (≈ 52 kDa)

Group	WIN-REP				HU-REP				VEH-REP					WIN-WD				HU-WD				VEH-WD			
Rat number	1	2	3	4	9	10	11	12	16	17	18	20	21	23	24	25	26	32	33	34	35	41	42	43	44

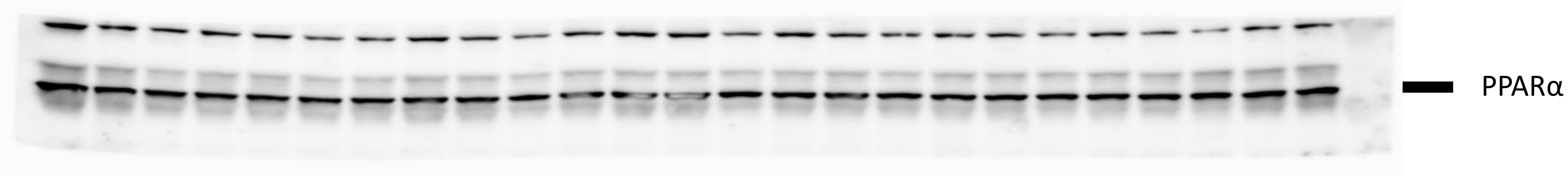

PPARα

Membrane 3: Immunoblot

Prefrontal cortex
PPARα (≈ 52 kDa)

Group	WIN-REP				HU-REP				VEH-REP					WIN-WD				HU-WD				VEH-WD			
Rat number	1	2	3	4	9	10	11	12	16	17	18	20	21	23	24	25	26	32	33	34	35	41	42	43	44

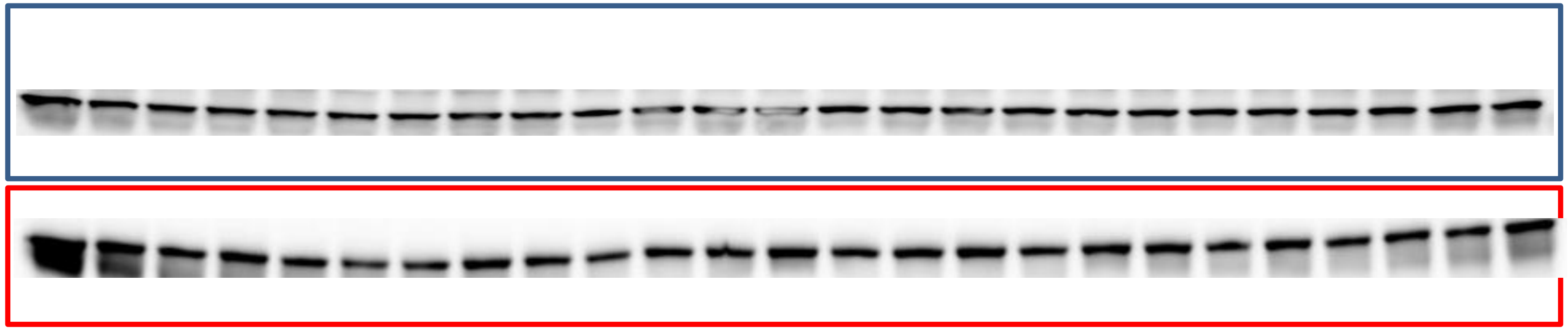

Membrane 3: Immunoblot

Prefrontal cortex
DAGLα (≈ 115 kDa)

Group	WIN-REP				HU-REP				VEH-REP					WIN-WD				HU-WD				VEH-WD			
Rat number	1	2	3	4	9	10	11	12	16	17	18	20	21	23	24	25	26	32	33	34	35	41	42	43	44

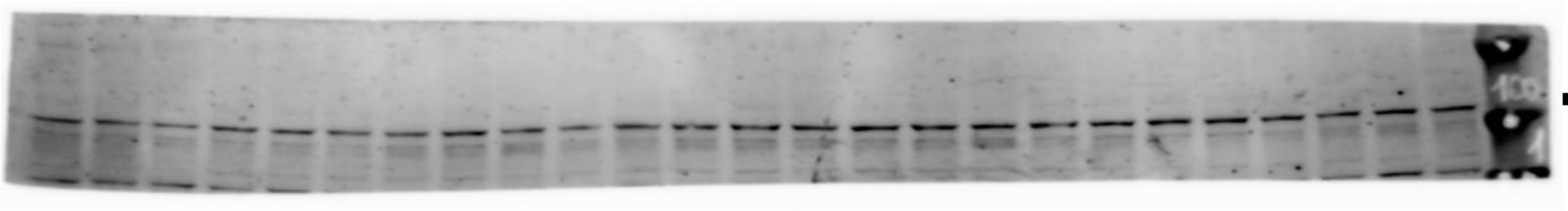

DAGLα

Membrane 3: Immunoblot

Prefrontal cortex
DAGLα (≈ 115 kDa)

Group	WIN-REP				HU-REP				VEH-REP					WIN-WD				HU-WD				VEH-WD			
Rat number	1	2	3	4	9	10	11	12	16	17	18	20	21	23	24	25	26	32	33	34	35	41	42	43	44

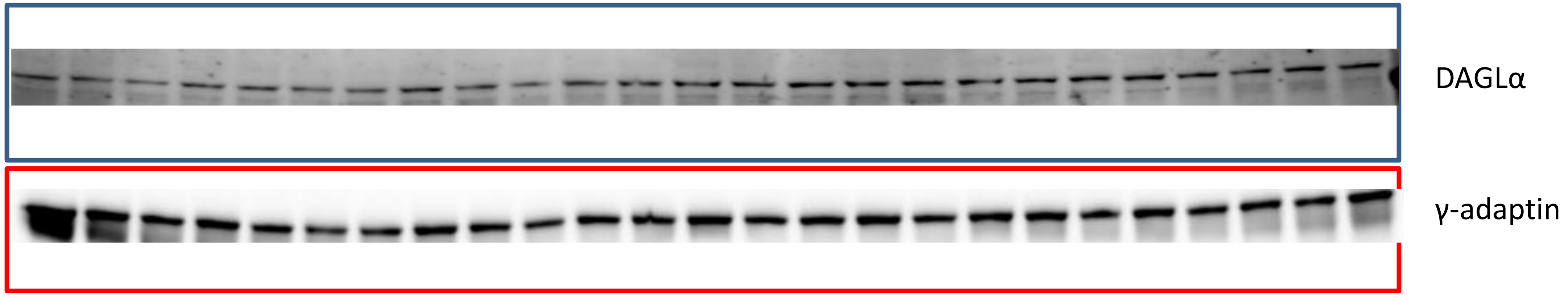

Membrane 3: Immunoblot

Prefrontal cortex
FAAH (≈ 63 kDa)

Group	WIN-REP				HU-REP				VEH-REP					WIN-WD				HU-WD				VEH-WD			
Rat number	1	2	3	4	9	10	11	12	16	17	18	20	21	23	24	25	26	32	33	34	35	41	42	43	44

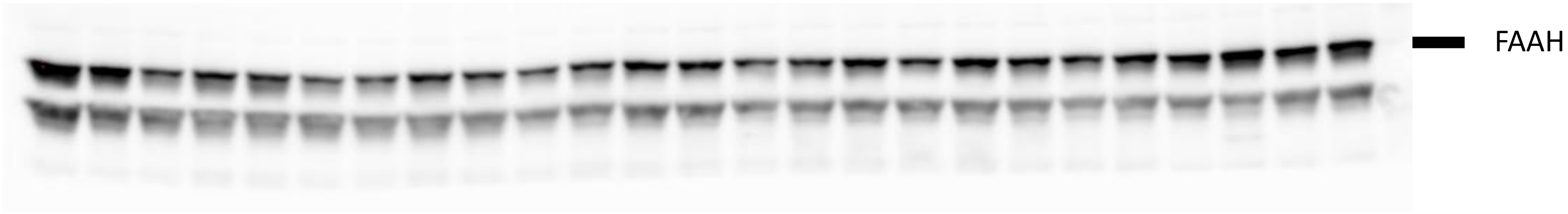

Membrane 3: Immunoblot

Prefrontal cortex
FAAH (≈ 63 kDa)

Group	WIN-REP				HU-REP				VEH-REP					WIN-WD				HU-WD				VEH-WD			
Rat number	1	2	3	4	9	10	11	12	16	17	18	20	21	23	24	25	26	32	33	34	35	41	42	43	44

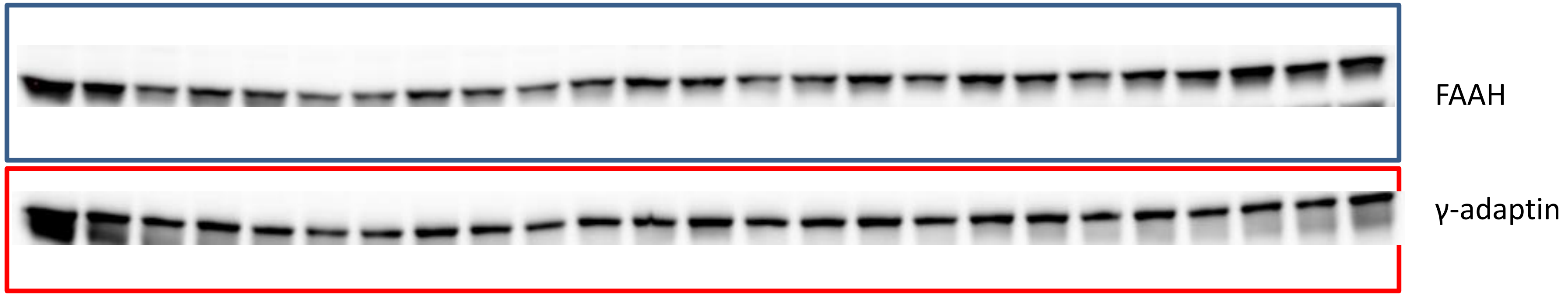

Gel 4: Ponceau S Red Staining

Prefrontal cortex

Main components (receptors and enzymes) of the endocannabinoid system: DAGLβ, NAPE-PLD, MAGL

Group	MW (kDa)	WIN-REP				HU-REP				VEH-REP					WIN-WD				HU-WD				VEH-WD			
Rat number		1	2	3	4	9	10	11	12	16	17	18	20	21	23	24	25	26	32	33	34	35	41	42	43	44

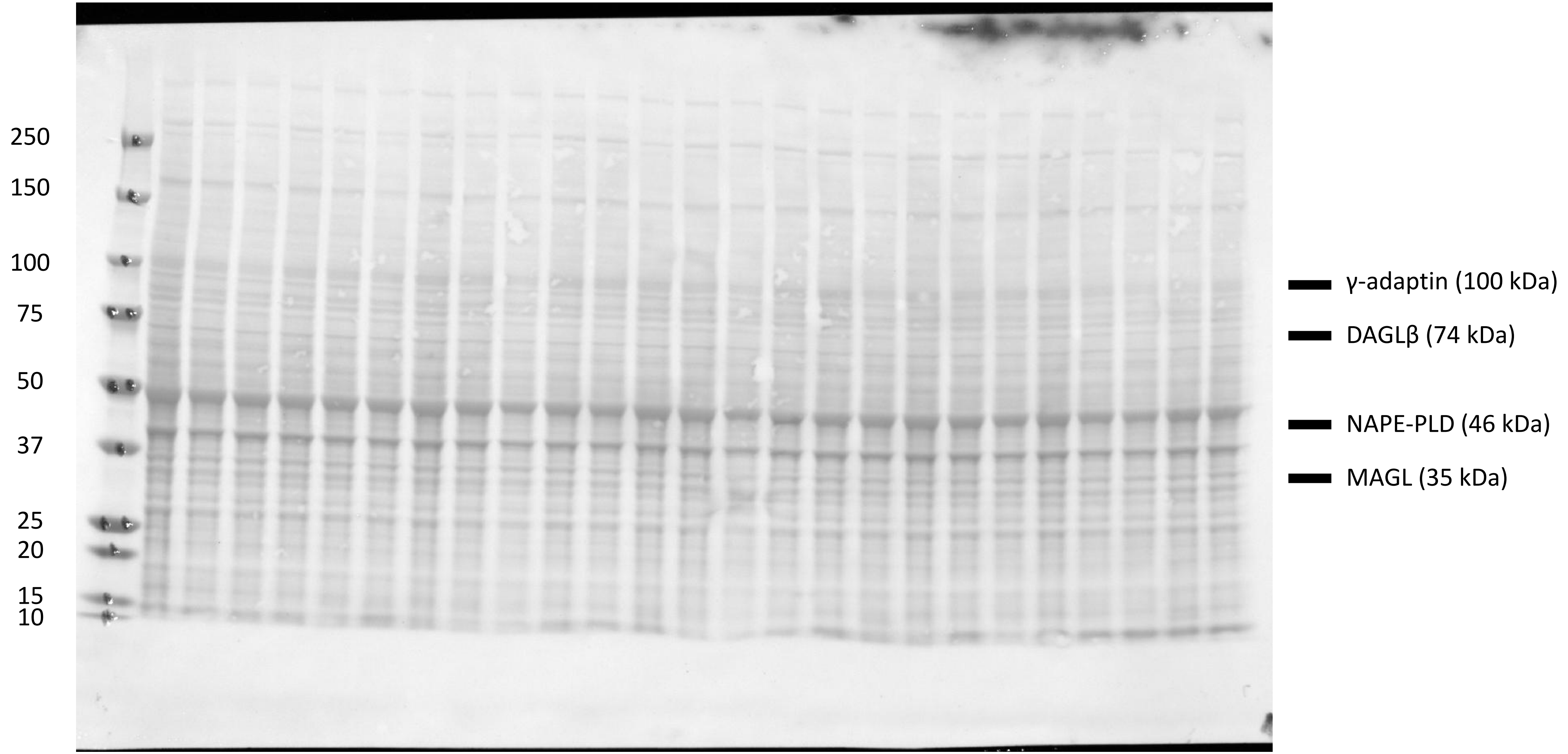

Membrane 4: Immunoblot

Prefrontal cortex
DAGLβ (≈ 74 kDa)

Group	WIN-REP				HU-REP				VEH-REP					WIN-WD				HU-WD				VEH-WD			
Rat number	1	2	3	4	9	10	11	12	16	17	18	20	21	23	24	25	26	32	33	34	35	41	42	43	44

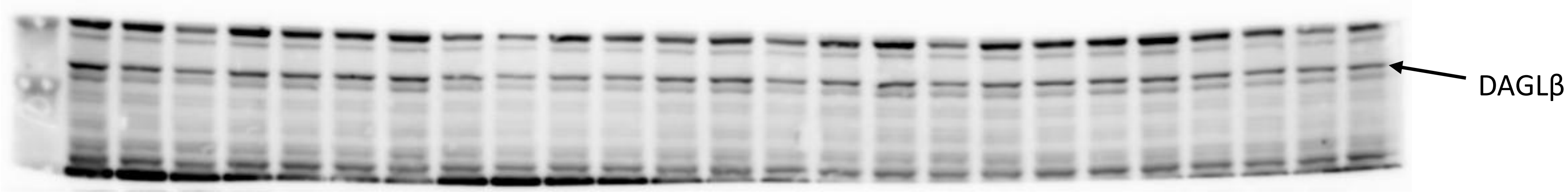

Membrane 4: Immunoblot

Prefrontal cortex
DAGLβ (≈ 74 kDa)

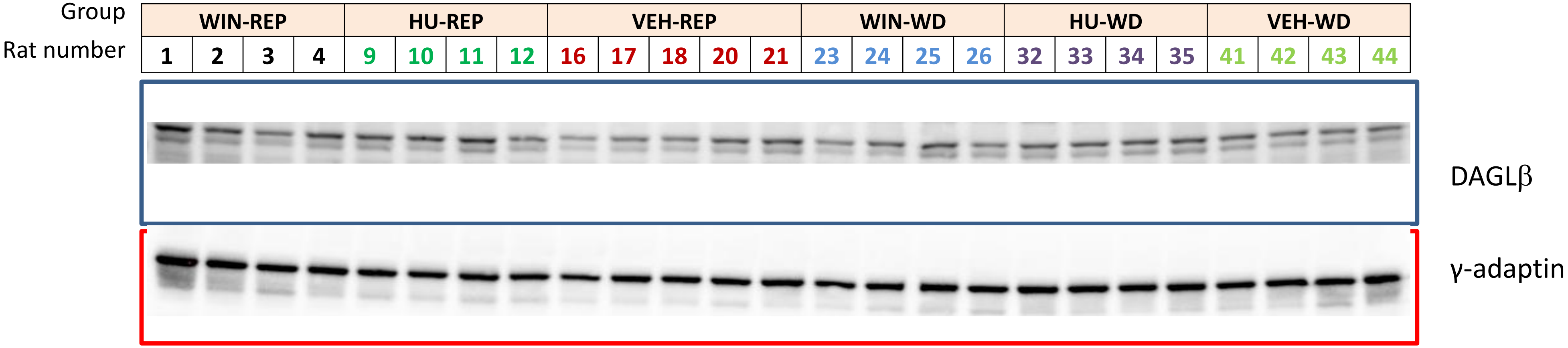

Membrane 4: Immunoblot

Prefrontal cortex
MAGL (≈ 35 kDa)

Group	WIN-REP				HU-REP				VEH-REP					WIN-WD				HU-WD				VEH-WD			
Rat number	1	2	3	4	9	10	11	12	16	17	18	20	21	23	24	25	26	32	33	34	35	41	42	43	44

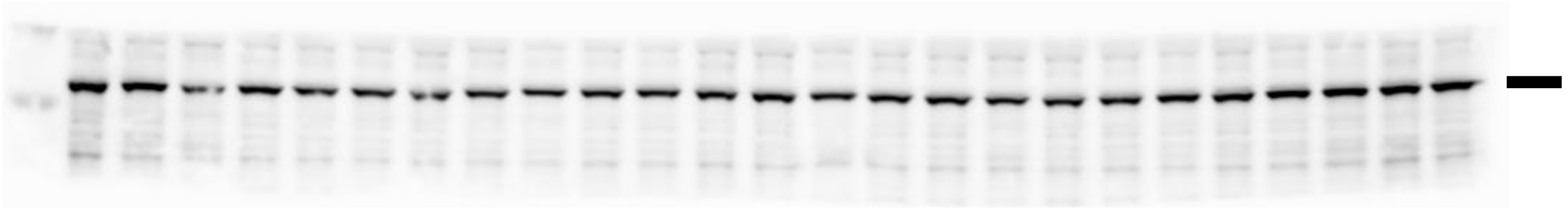

MAGL

Membrane 4: Immunoblot

Prefrontal cortex
MAGL (≈ 35 kDa)

Group	WIN-REP				HU-REP				VEH-REP					WIN-WD				HU-WD				VEH-WD			
Rat number	1	2	3	4	9	10	11	12	16	17	18	20	21	23	24	25	26	32	33	34	35	41	42	43	44

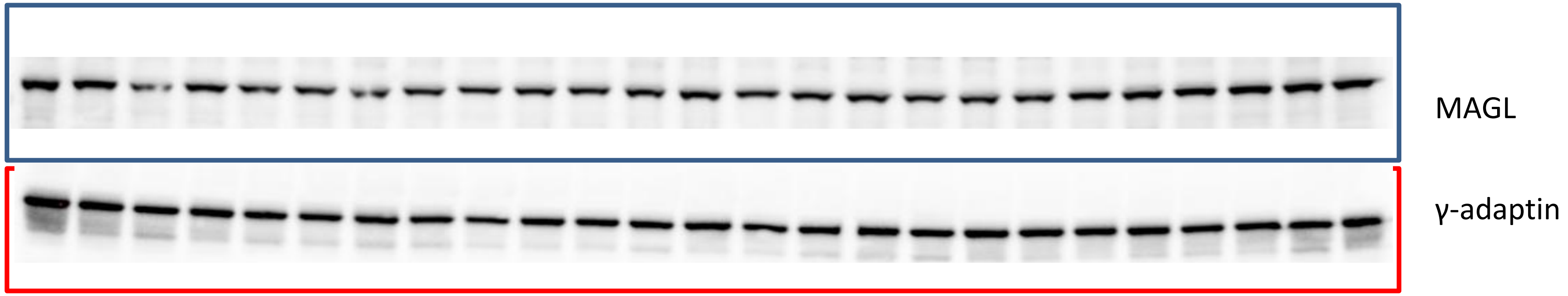

Membrane 4: Immunoblot

Prefrontal cortex
NAPE-PLD (≈ 46 kDa)

Group	WIN-REP				HU-REP				VEH-REP					WIN-WD				HU-WD				VEH-WD			
Rat number	1	2	3	4	9	10	11	12	16	17	18	20	21	23	24	25	26	32	33	34	35	41	42	43	44

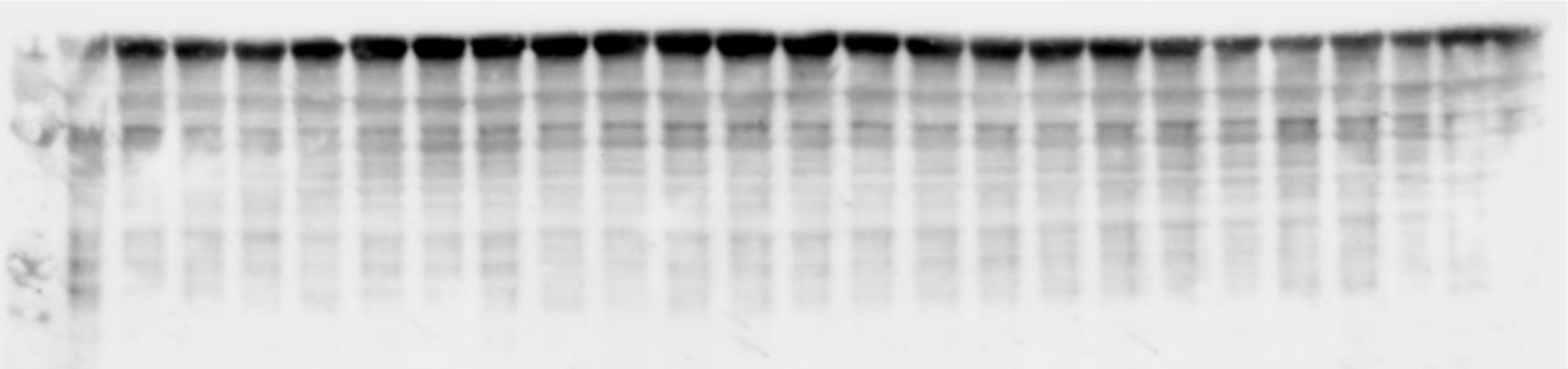

NAPE-PLD

Membrane 4: Immunoblot

Prefrontal cortex
NAPE-PLD (≈ 46 kDa)

Group	WIN-REP				HU-REP				VEH-REP					WIN-WD				HU-WD				VEH-WD			
Rat number	1	2	3	4	9	10	11	12	16	17	18	20	21	23	24	25	26	32	33	34	35	41	42	43	44

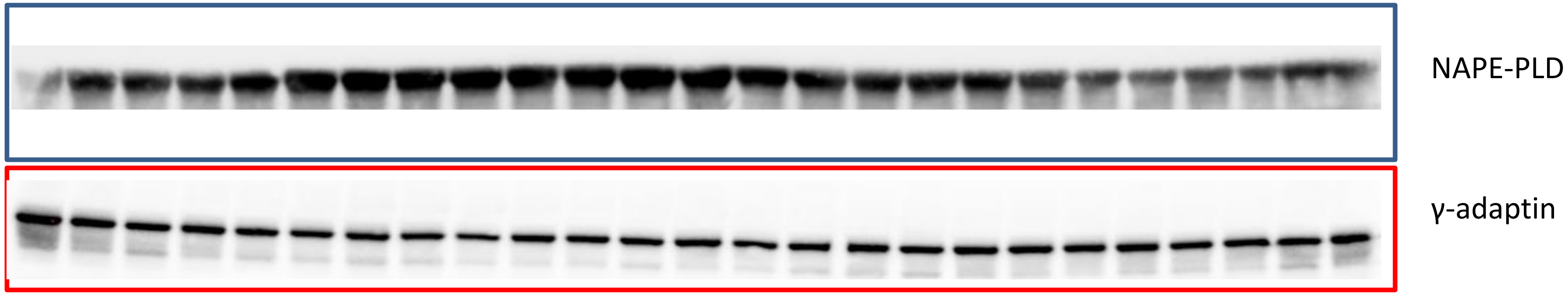

Gel 5: Ponceau S Red Staining

Prefrontal cortex
Biomarkers of glial cells: GFAP, IBA1, vimentin

Group	MW (kDa)	WIN-REP				HU-REP				VEH-REP					WIN-WD				HU-WD				VEH-WD			
Rat number		1	2	3	4	9	10	11	12	16	17	18	20	21	23	24	25	26	32	33	34	35	41	42	43	44

missing

Membrane 5: Immunoblot

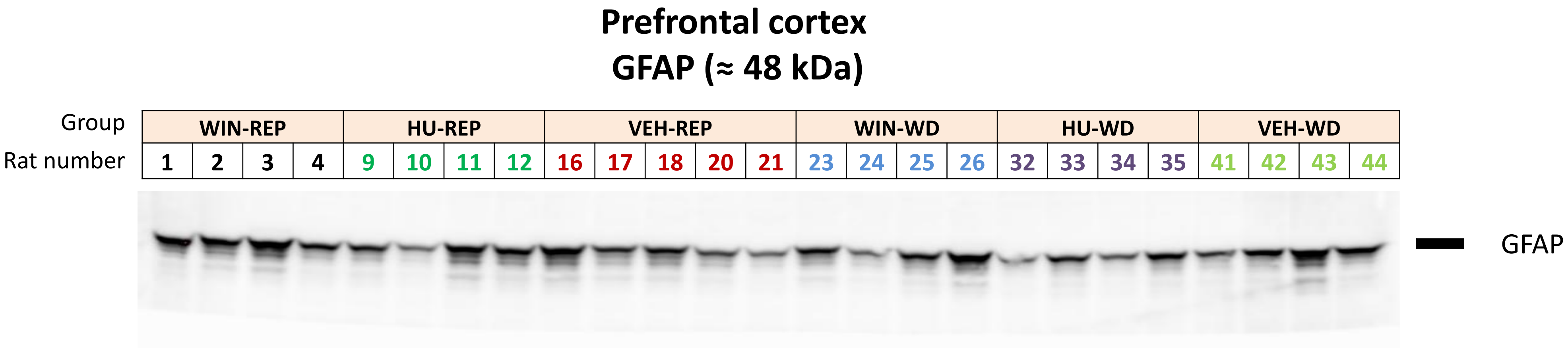

Membrane 5: Immunoblot

Prefrontal cortex
GFAP (≈ 48 kDa)

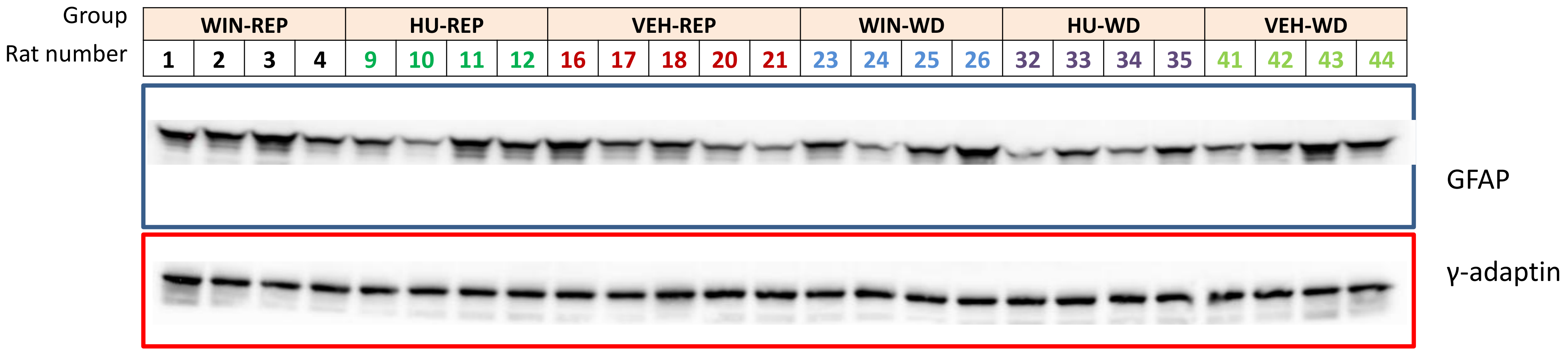

Membrane 5: Immunoblot

Prefrontal cortex
IBA1 (≈ 17 kDa)

Group	WIN-REP				HU-REP				VEH-REP					WIN-WD				HU-WD				VEH-WD			
Rat number	1	2	3	4	9	10	11	12	16	17	18	20	21	23	24	25	26	32	33	34	35	41	42	43	44

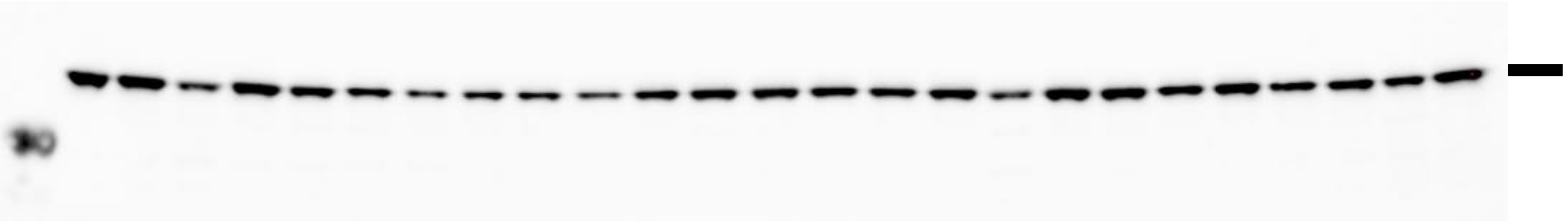

IBA1

Membrane 5: Immunoblot

Prefrontal cortex
IBA1 (≈ 17 kDa)

Group	WIN-REP				HU-REP				VEH-REP					WIN-WD				HU-WD				VEH-WD			
Rat number	1	2	3	4	9	10	11	12	16	17	18	20	21	23	24	25	26	32	33	34	35	41	42	43	44

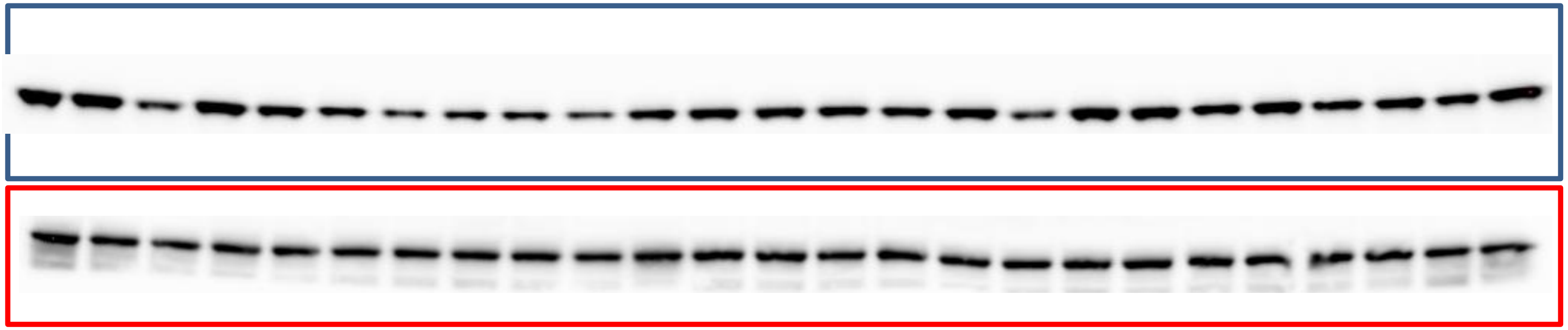

IBA1

γ-adaptin

Membrane 5: Immunoblot

Prefrontal cortex
Vimentin (≈ 54 kDa)

Group	WIN-REP				HU-REP				VEH-REP					WIN-WD				HU-WD				VEH-WD			
Rat number	1	2	3	4	9	10	11	12	16	17	18	20	21	23	24	25	26	32	33	34	35	41	42	43	44

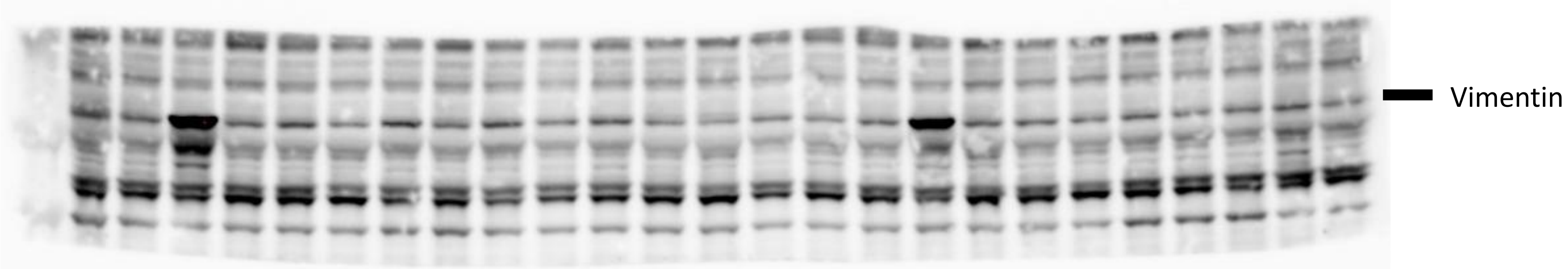

Membrane 5: Immunoblot

Prefrontal cortex
Vimentin (≈ 54 kDa)

Group	WIN-REP				HU-REP				VEH-REP					WIN-WD				HU-WD				VEH-WD			
Rat number	1	2	3	4	9	10	11	12	16	17	18	20	21	23	24	25	26	32	33	34	35	41	42	43	44

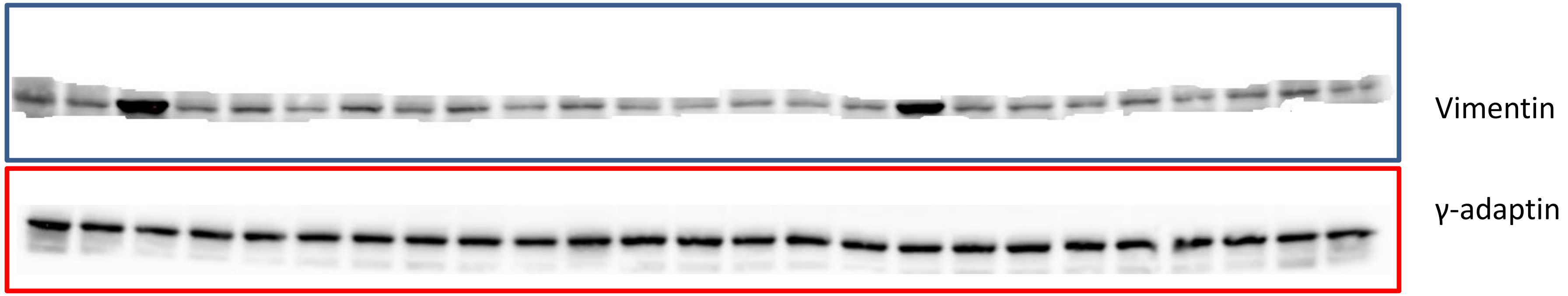

Gel 6: Ponceau S Red Staining

Prefrontal cortex
Factors involved in neuroinflammation: COX2

Group	MW (kDa)	WIN-REP				HU-REP				VEH-REP					WIN-WD				HU-WD				VEH-WD			
Rat number		1	2	3	4	9	10	11	12	16	17	18	20	21	23	24	25	26	32	33	34	35	41	42	43	44

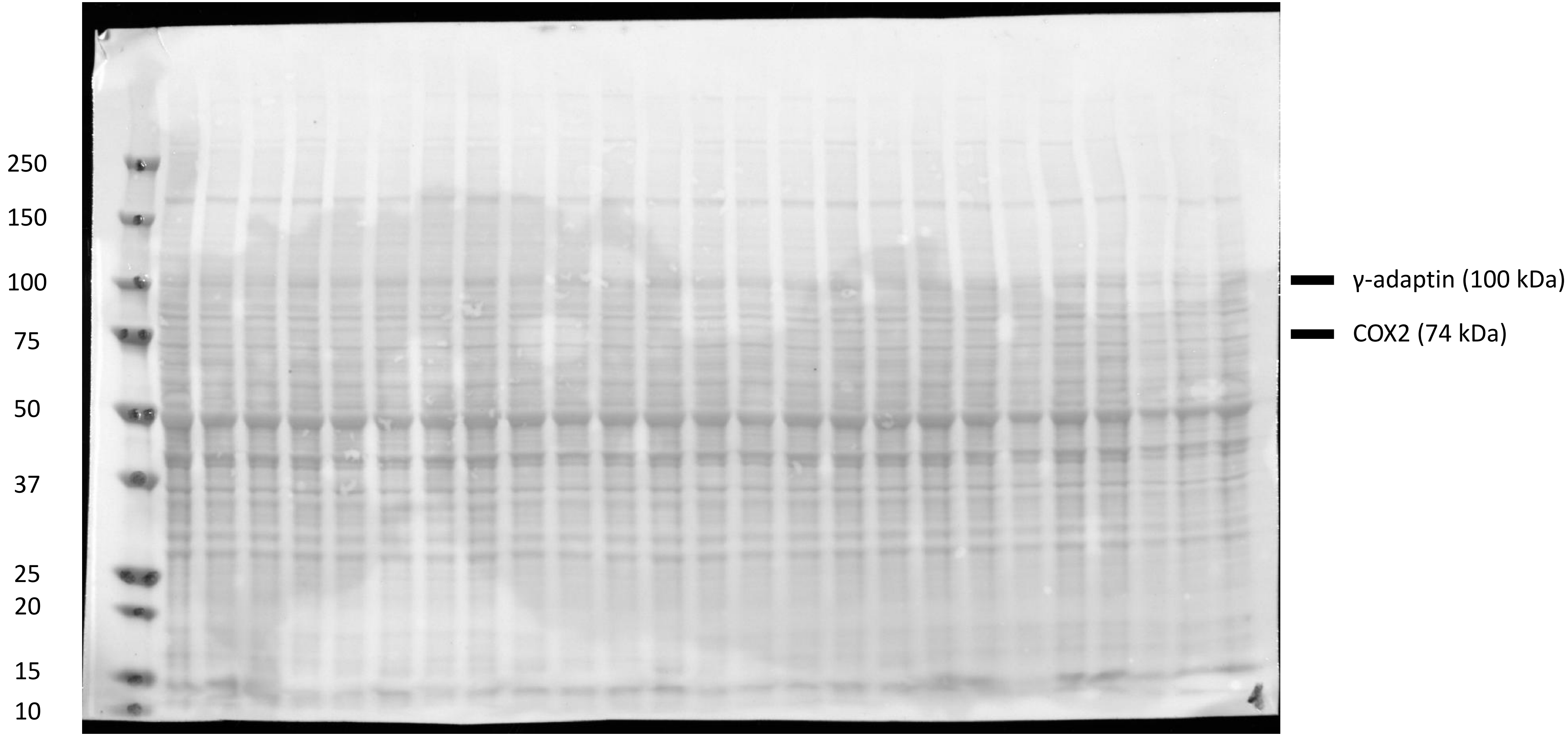

Membrane 6: Immunoblot

Prefrontal cortex
COX2 (≈ 74 kDa)

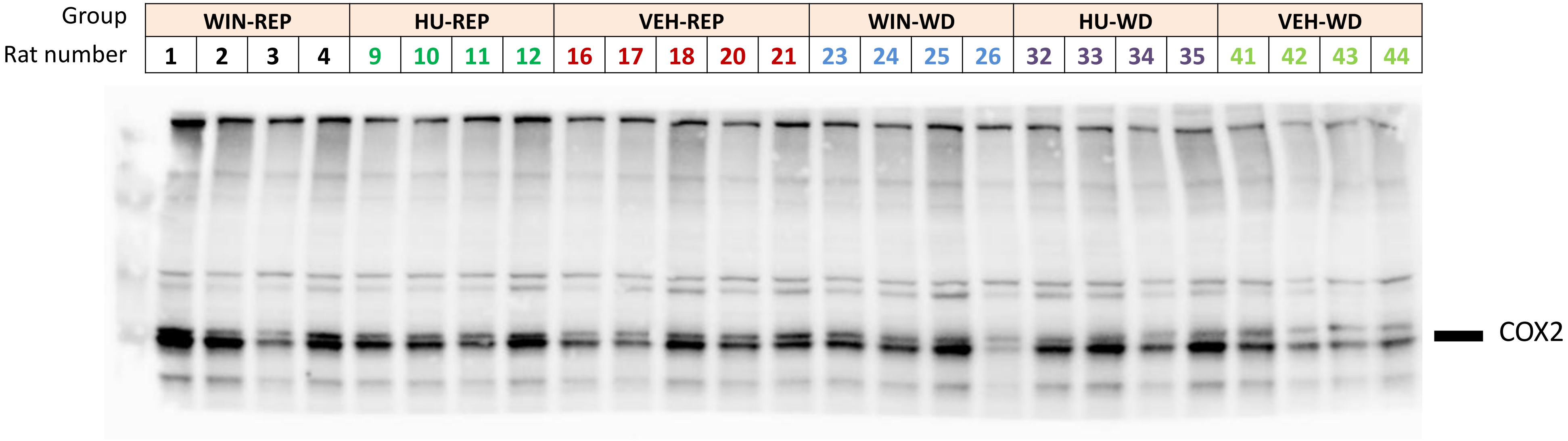

Membrane 6: Immunoblot

Prefrontal cortex

COX2 (≈ 74 kDa)

Group	WIN-REP				HU-REP				VEH-REP					WIN-WD				HU-WD				VEH-WD			
Rat number	1	2	3	4	9	10	11	12	16	17	18	20	21	23	24	25	26	32	33	34	35	41	42	43	44

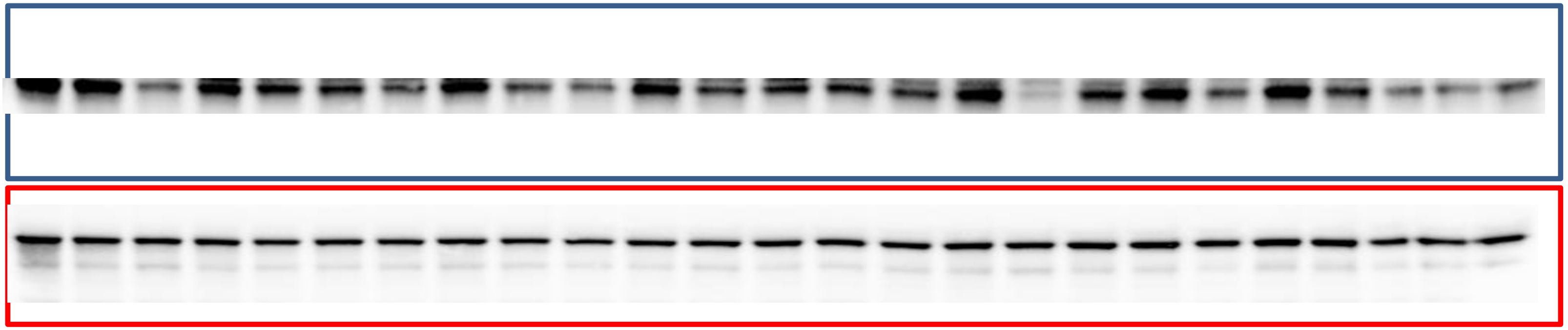

COX2

γ-adaptin

Gel 7: Ponceau S Red Staining

Prefrontal cortex
Factors involved in neuroinflammation: IKKβ, NF-κB

Group	WIN-REP				HU-REP				VEH-REP					WIN-WD				HU-WD				VEH-WD				MW (kDa)
Rat number	1	2	3	4	9	10	11	12	16	17	18	20	21	23	24	25	26	32	33	34	35	41	42	43	44	

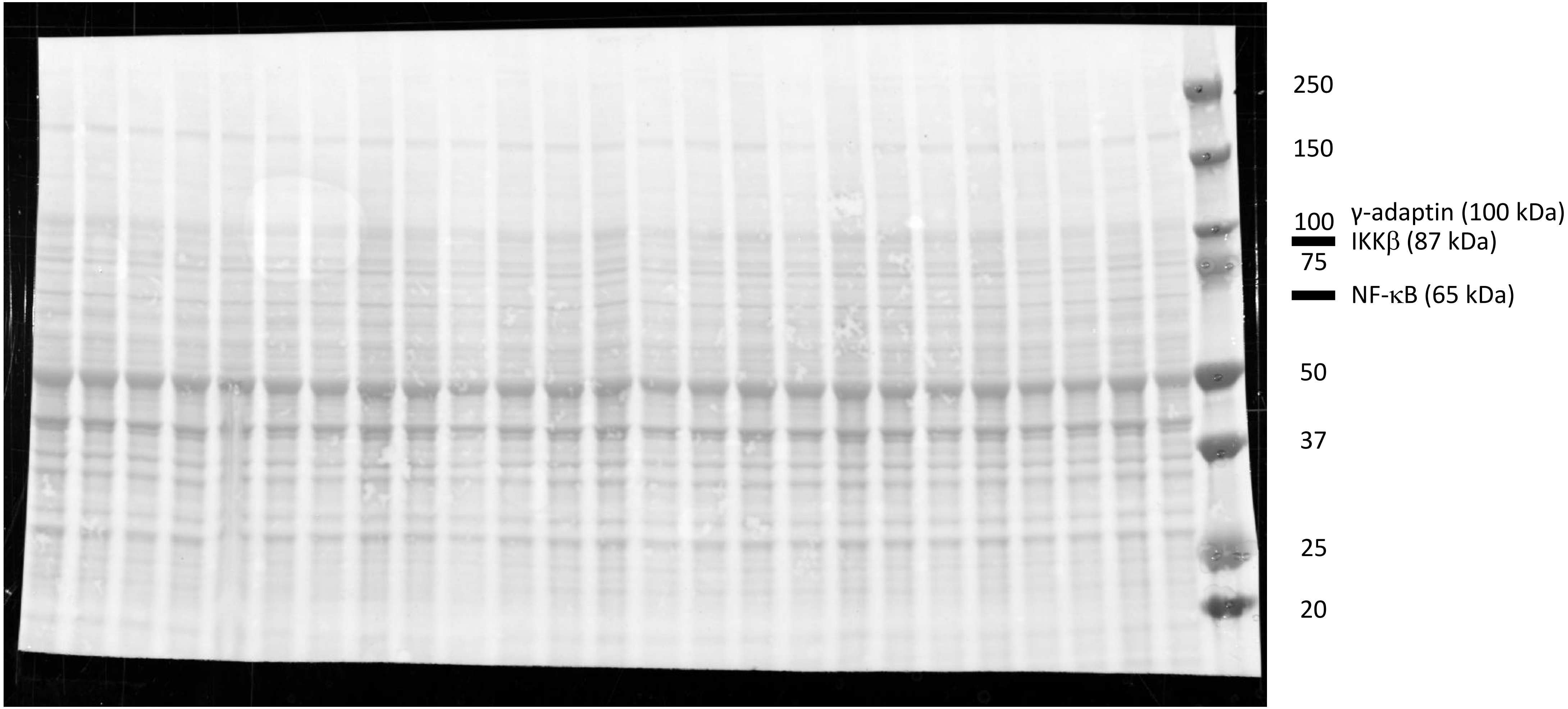

Membrane 7: Immunoblot

Prefrontal cortex
NF-κB (≈ 65 kDa)

Group	WIN-REP				HU-REP				VEH-REP					WIN-WD				HU-WD				VEH-WD			
Rat number	1	2	3	4	9	10	11	12	16	17	18	20	21	23	24	25	26	32	33	34	35	41	42	43	44

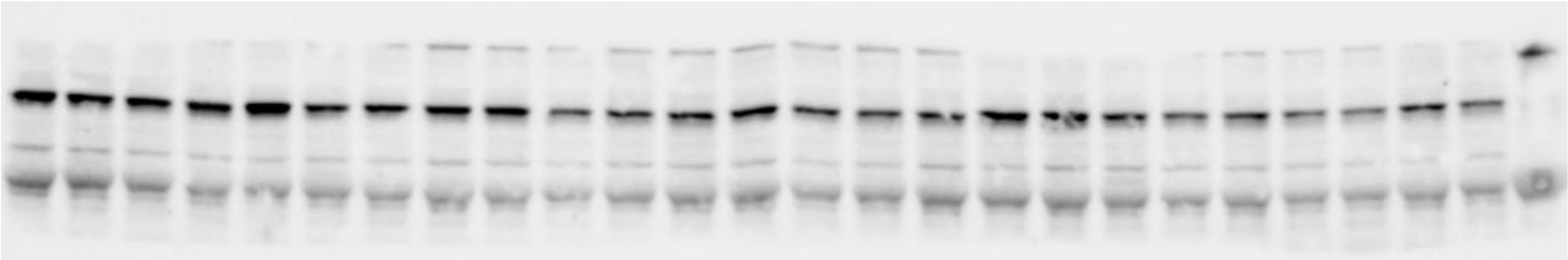

NF-κB

Membrane 7: Immunoblot

Prefrontal cortex

NF-κB (≈ 65 kDa)

Group	WIN-REP				HU-REP				VEH-REP					WIN-WD				HU-WD				VEH-WD			
Rat number	1	2	3	4	9	10	11	12	16	17	18	20	21	23	24	25	26	32	33	34	35	41	42	43	44

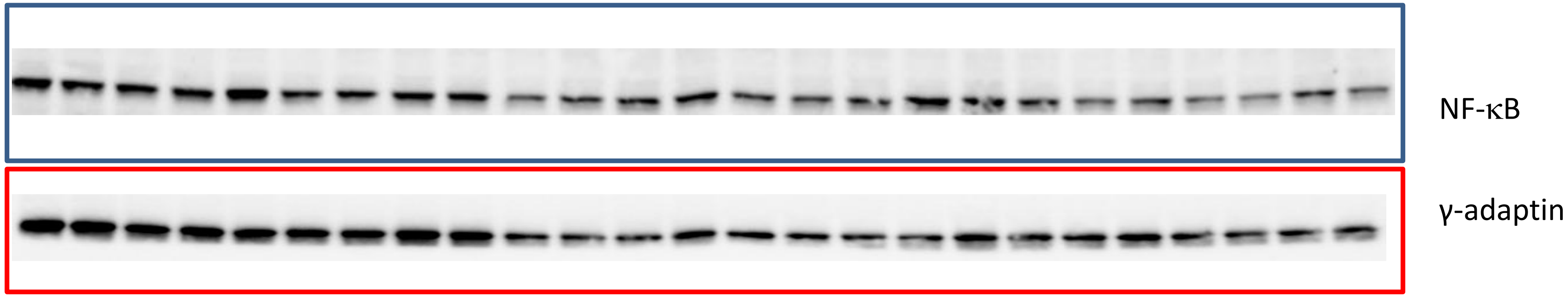

Membrane 7: Immunoblot

Prefrontal cortex
IKKβ (≈ 87 kDa)

Group	WIN-REP				HU-REP				VEH-REP					WIN-WD				HU-WD				VEH-WD			
Rat number	1	2	3	4	9	10	11	12	16	17	18	20	21	23	24	25	26	32	33	34	35	41	42	43	44

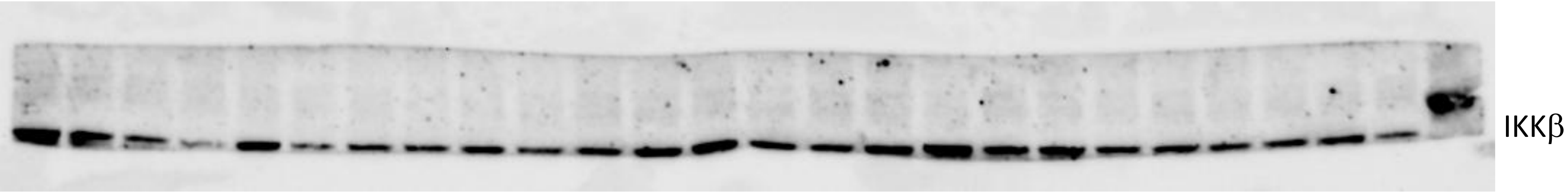

Membrane 7: Immunoblot

Prefrontal cortex
IKKβ (≈ 87 kDa)

Group	WIN-REP				HU-REP				VEH-REP					WIN-WD				HU-WD				VEH-WD			
Rat number	1	2	3	4	9	10	11	12	16	17	18	20	21	23	24	25	26	32	33	34	35	41	42	43	44

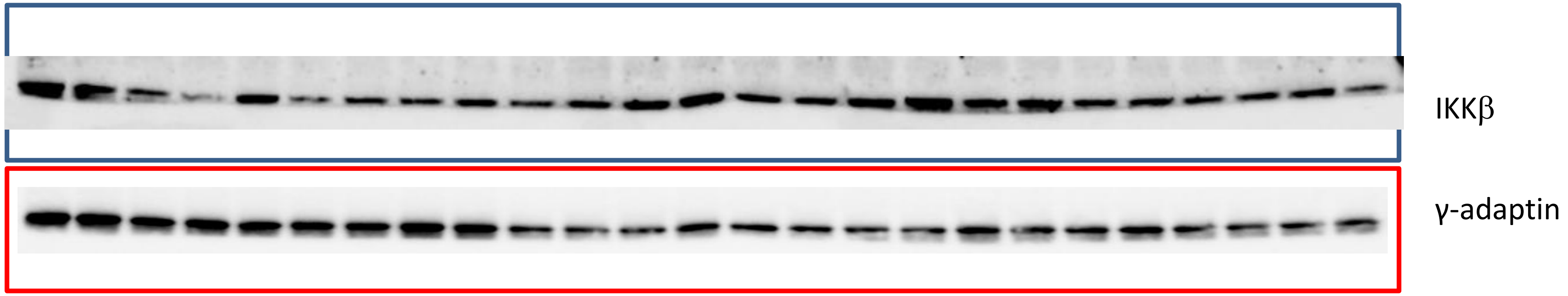

Summary of immunoblots (see Figure 2)

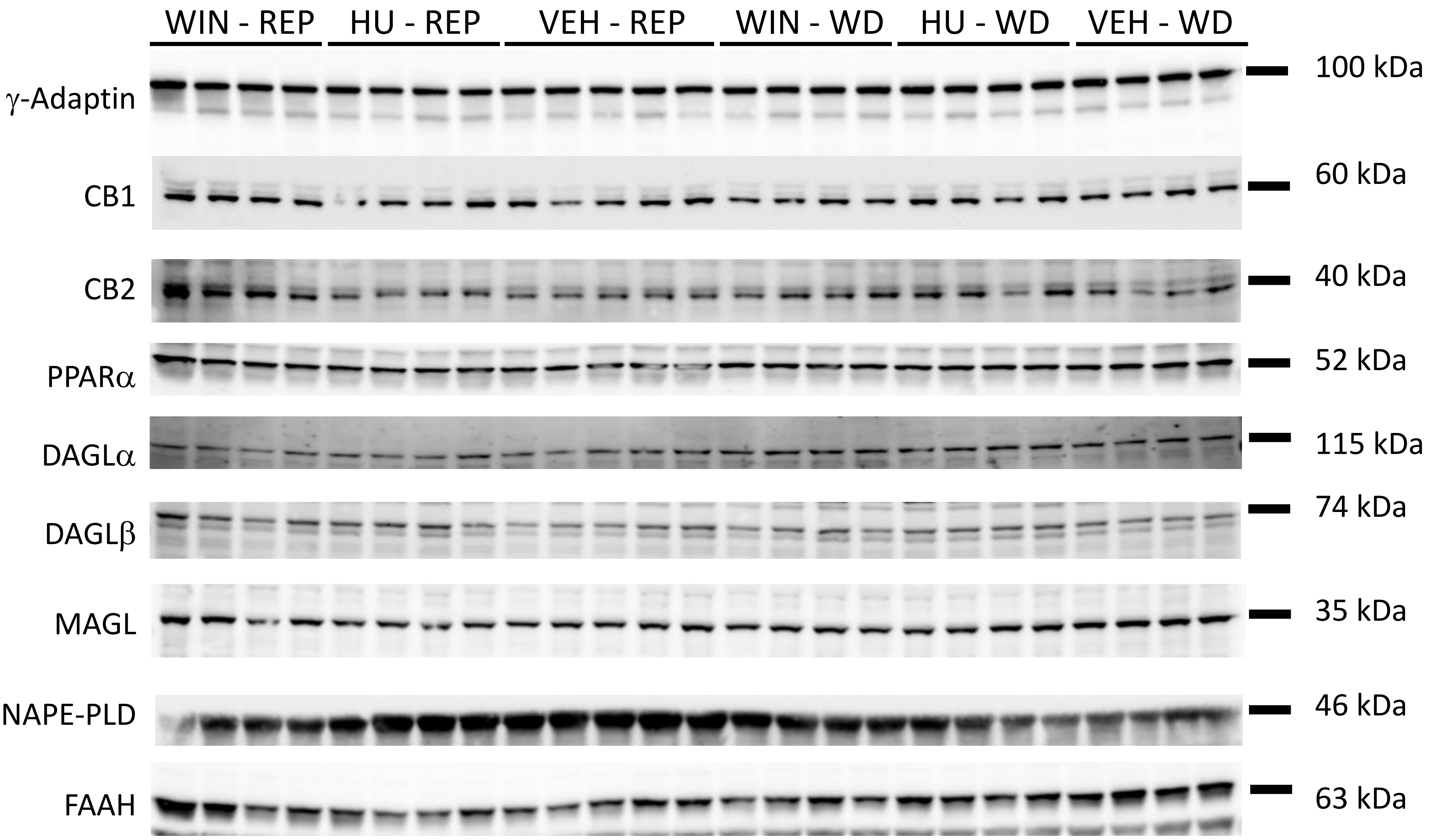

Summary of immunoblots (see Figure 3)

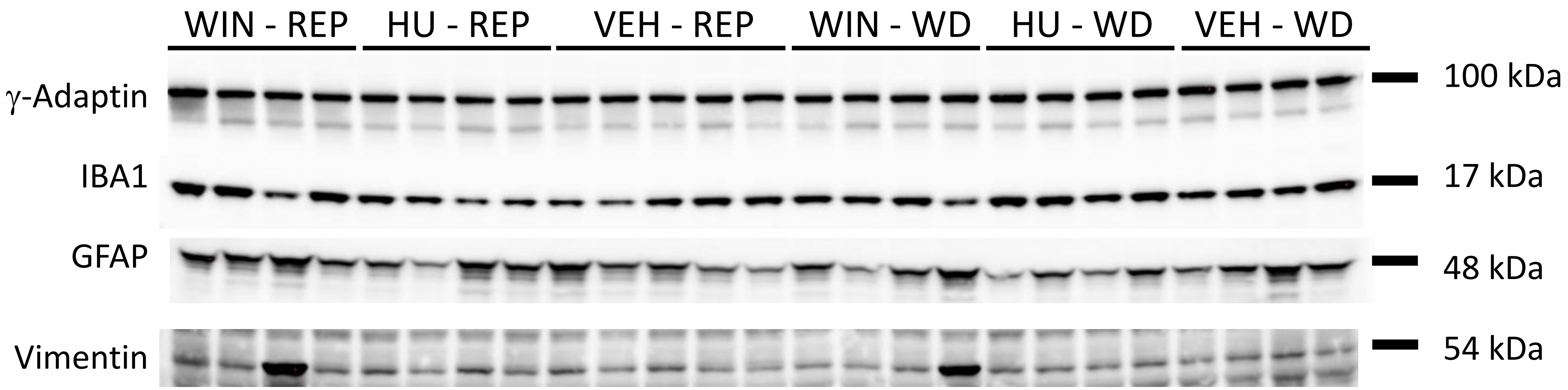

Summary of immunoblots (see Figure 3)

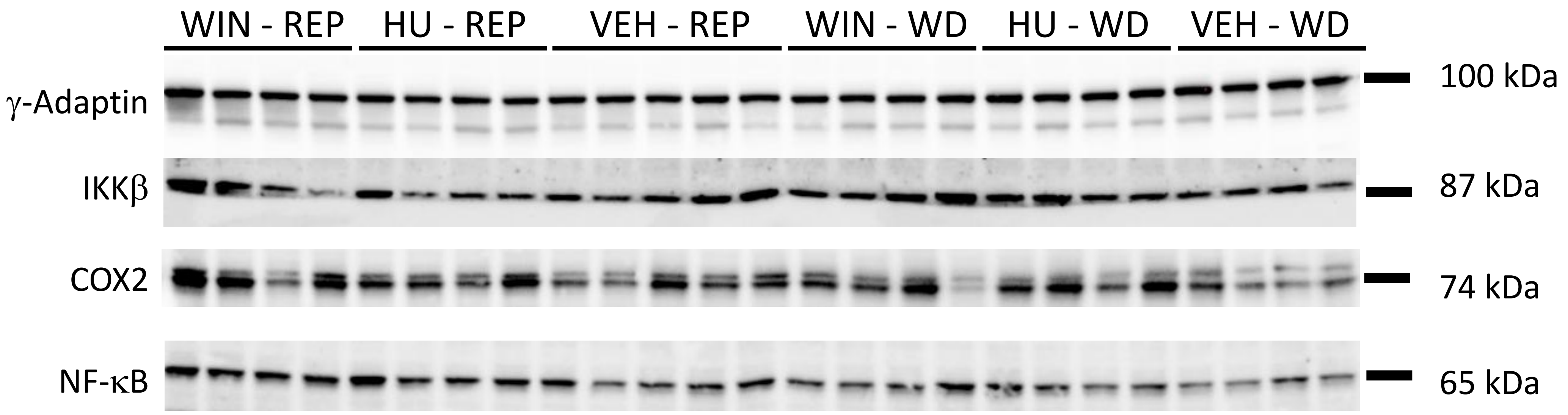

Supplementary Figure S2

Western Blots
Gels and immunoblots
Hippocampus

…involving the hippocampus of Wistar rats treated with WIN 55,212-2, HU-210, and a vehicle under conditions of repeated administration and withdrawal.

Each lane represents a sample from one rat, with the following groups:

- **WIN 55,212-2 Repeated administration**: n = 4
- **HU-210 Repeated administration**: n = 4
- **Vehicle Repeated administration**: n = 5
- **WIN 55,212-2 Withdrawal**: n = 4
- **HU-210 Withdrawal**: n = 4
- **Vehicle Withdrawal**: n = 4

Gel 1: Ponceau S Red Staining

Hippocampus

Main components (receptors and enzymes) of the endocannabinoid system: CB1

Group	MW (kDa)	WIN-REP				HU-REP				VEH-REP					WIN-WD				HU-WD				VEH-WD			
Rat number		1	2	3	4	9	10	11	12	16	17	18	20	21	23	24	25	26	32	33	34	35	41	42	43	44

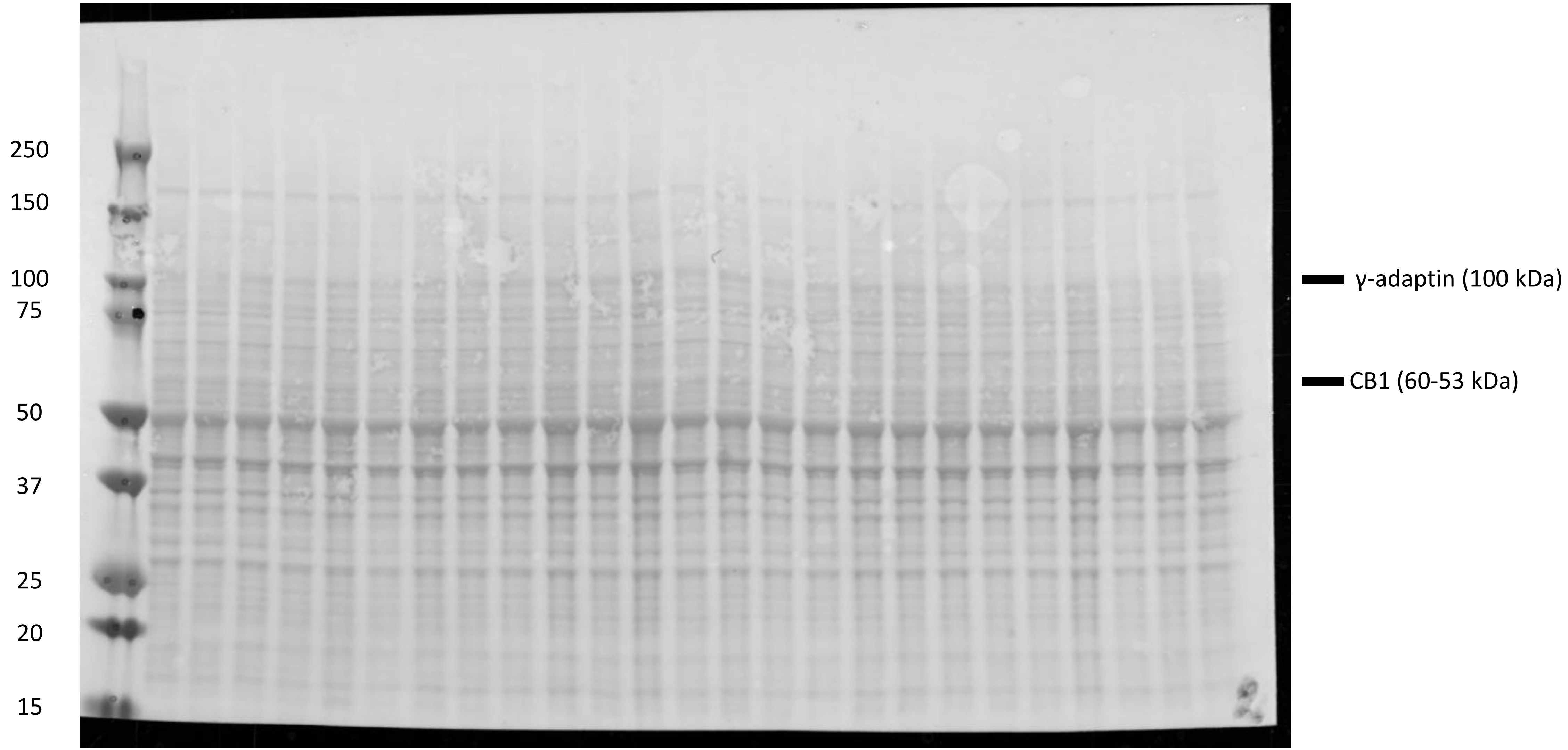

Membrane 1: Immunoblot

Hippocampus
CB1 (≈ 53-60 kDa)

Group	WIN-REP				HU-REP				VEH-REP					WIN-WD				HU-WD				VEH-WD			
Rat number	1	2	3	4	9	10	11	12	16	17	18	20	21	23	24	25	26	32	33	34	35	41	42	43	44

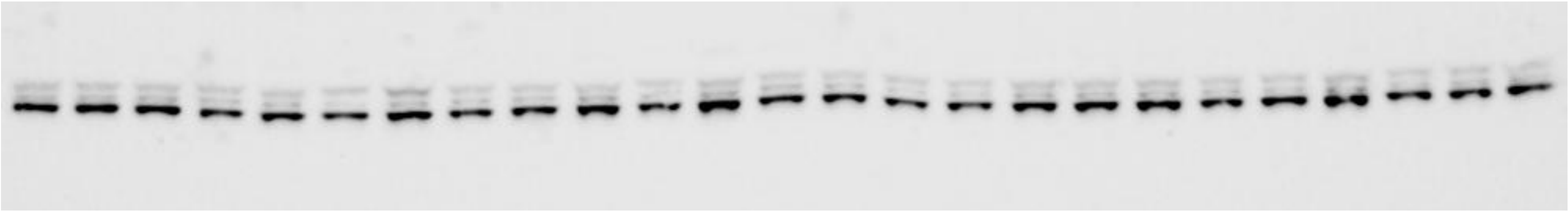

CB1

Membrane 1: Immunoblot

Seeman Hippocampus
CB1 (≈ 53-60 kDa)

Group	WIN-REP				HU-REP				VEH-REP					WIN-WD				HU-WD				VEH-WD			
Rat number	1	2	3	4	9	10	11	12	16	17	18	20	21	23	24	25	26	32	33	34	35	41	42	43	44

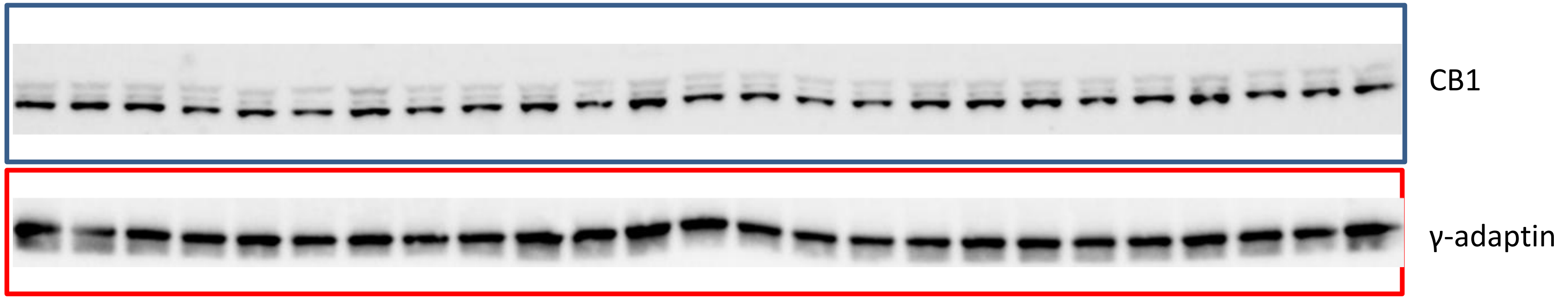

Gel 2: Ponceau S Red Staining

Hippocampus

Main components (receptors and enzymes) of the endocannabinoid system: CB2

Group	MW (kDa)	WIN-REP				HU-REP				VEH-REP					WIN-WD				HU-WD				VEH-WD			
Rat number		1	2	3	4	9	10	11	12	16	17	18	20	21	23	24	25	26	32	33	34	35	41	42	43	44

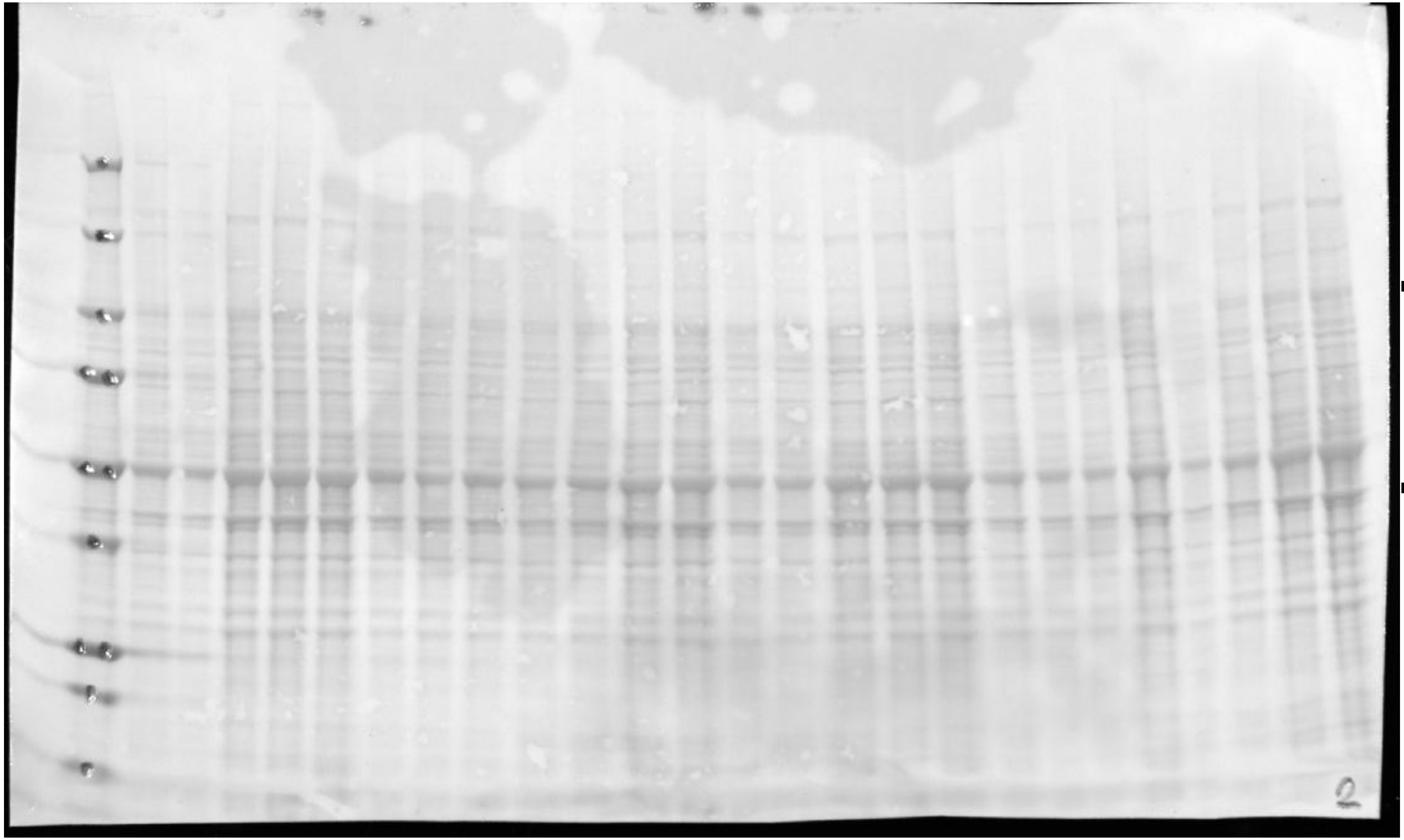

250
150
100
75
50
37
25
20
15

γ-adaptin (100 kDa)

CB2 (40 kDa)

Membrane 2: Immunoblot

Hippocampus
CB2 (≈ 40 kDa)

Group	WIN-REP				HU-REP				VEH-REP					WIN-WD				HU-WD				VEH-WD			
Rat number	1	2	3	4	9	10	11	12	16	17	18	20	21	23	24	25	26	32	33	34	35	41	42	43	44

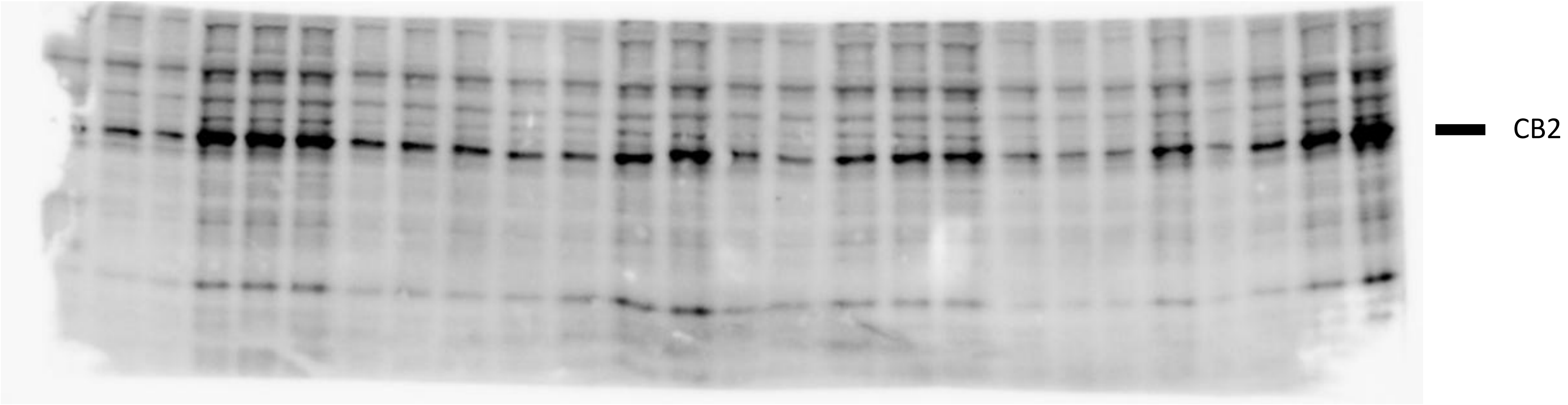

Membrane 2: Immunoblot

Hippocampus
CB2 (≈ 40 kDa)

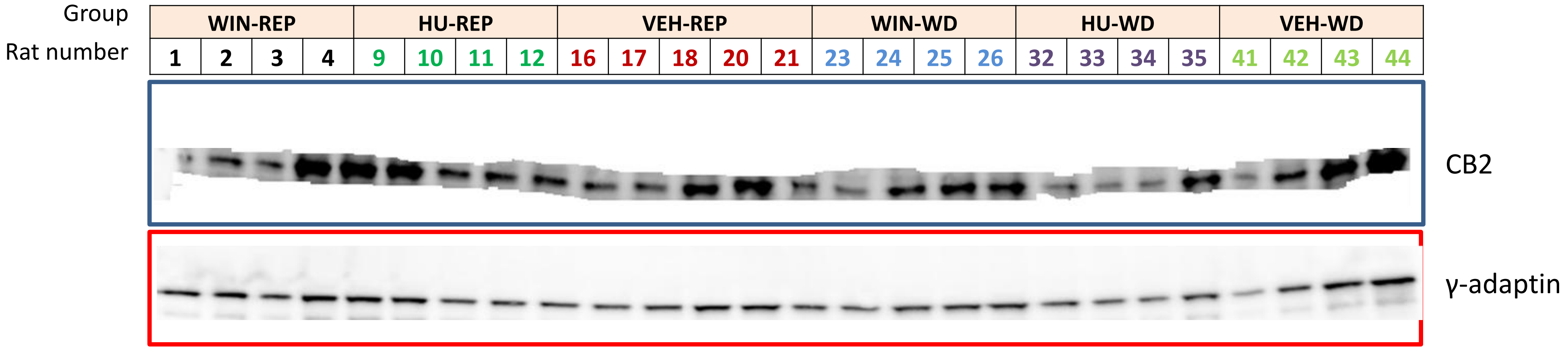

Gel 3: Ponceau S Red Staining

Hippocampus

Main components (receptors and enzymes) of the endocannabinoid system: DAGLα, PPARα, FAAH

Group	MW (kDa)	WIN-REP				HU-REP				VEH-REP					WIN-WD				HU-WD				VEH-WD			
Rat number		1	2	3	4	9	10	11	12	16	17	18	20	21	23	24	25	26	32	33	34	35	41	42	43	44

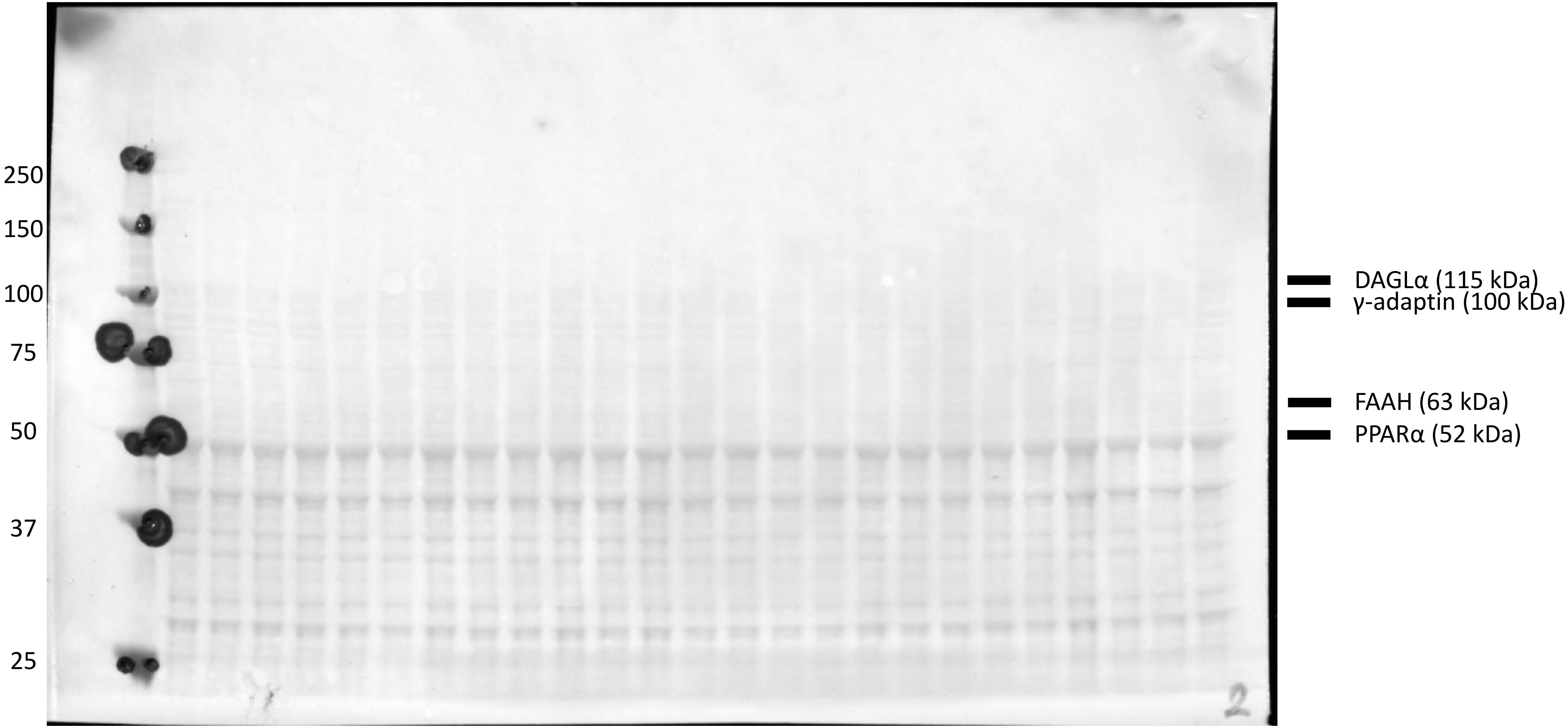

Membrane 3: Immunoblot

Hippocampus
PPARα (≈ 52 kDa)

Group	WIN-REP				HU-REP				VEH-REP					WIN-WD				HU-WD				VEH-WD			
Rat number	1	2	3	4	9	10	11	12	16	17	18	20	21	23	24	25	26	32	33	34	35	41	42	43	44

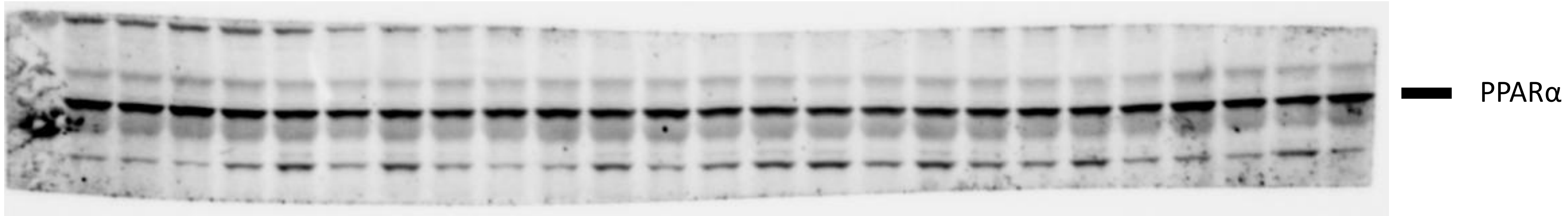

Membrane 3: Immunoblot

Hippocampus
PPARα (≈ 52 kDa)

Group	WIN-REP				HU-REP				VEH-REP					WIN-WD				HU-WD				VEH-WD			
Rat number	1	2	3	4	9	10	11	12	16	17	18	20	21	23	24	25	26	32	33	34	35	41	42	43	44

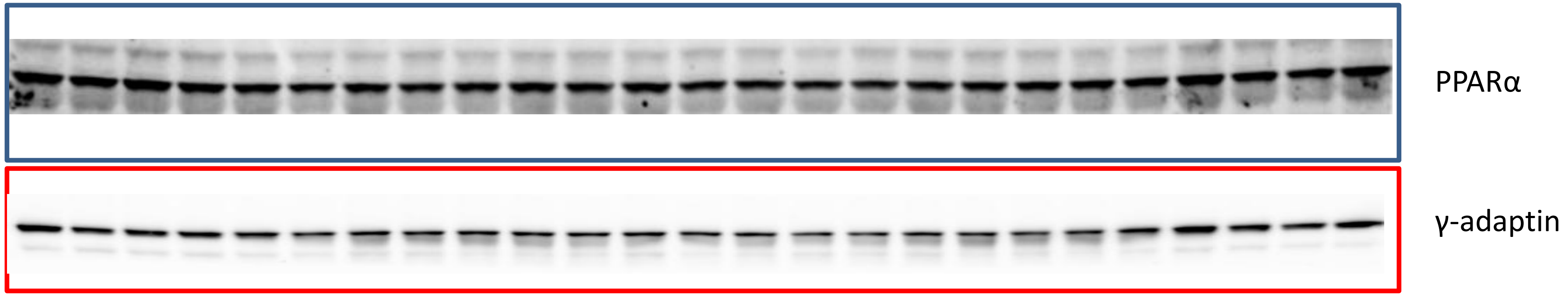

Membrane 3: Immunoblot

Hippocampus
DAGLα (≈ 115 kDa)

Group	WIN-REP				HU-REP				VEH-REP					WIN-WD				HU-WD				VEH-WD			
Rat number	1	2	3	4	9	10	11	12	16	17	18	20	21	23	24	25	26	32	33	34	35	41	42	43	44

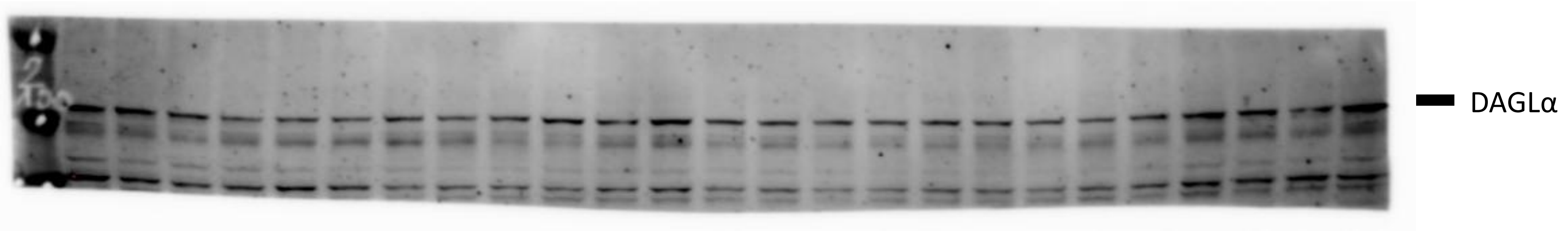

Membrane 3: Immunoblot

Hippocampus
DAGLα (≈ 115 kDa)

Group	WIN-REP				HU-REP				VEH-REP					WIN-WD				HU-WD				VEH-WD			
Rat number	1	2	3	4	9	10	11	12	16	17	18	20	21	23	24	25	26	32	33	34	35	41	42	43	44

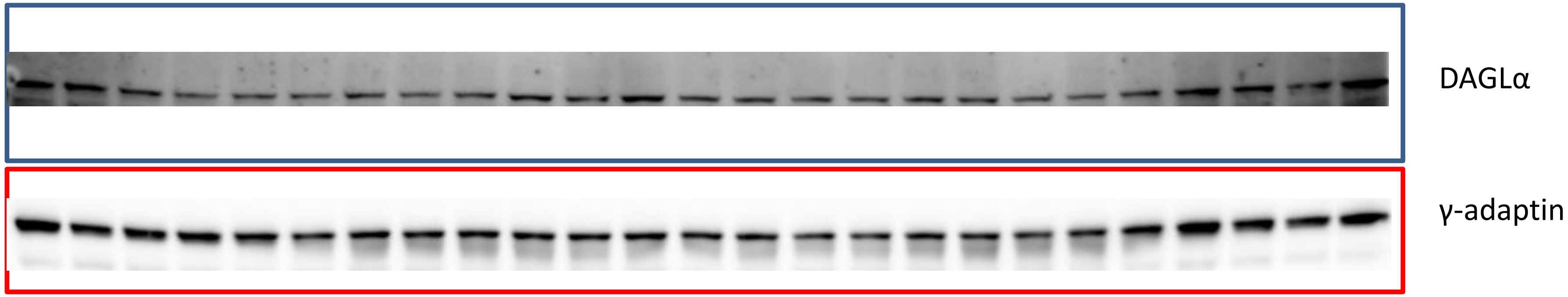

Membrane 3: Immunoblot

Hippocampus
FAAH (≈ 63 kDa)

Group	WIN-REP				HU-REP				VEH-REP					WIN-WD				HU-WD				VEH-WD			
Rat number	1	2	3	4	9	10	11	12	16	17	18	20	21	23	24	25	26	32	33	34	35	41	42	43	44

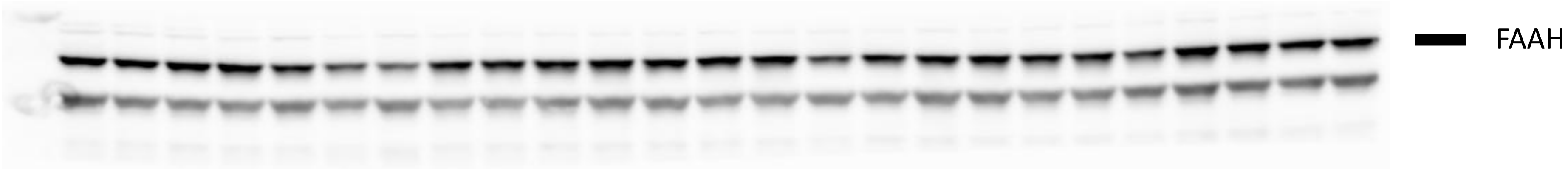

Membrane 3: Immunoblot

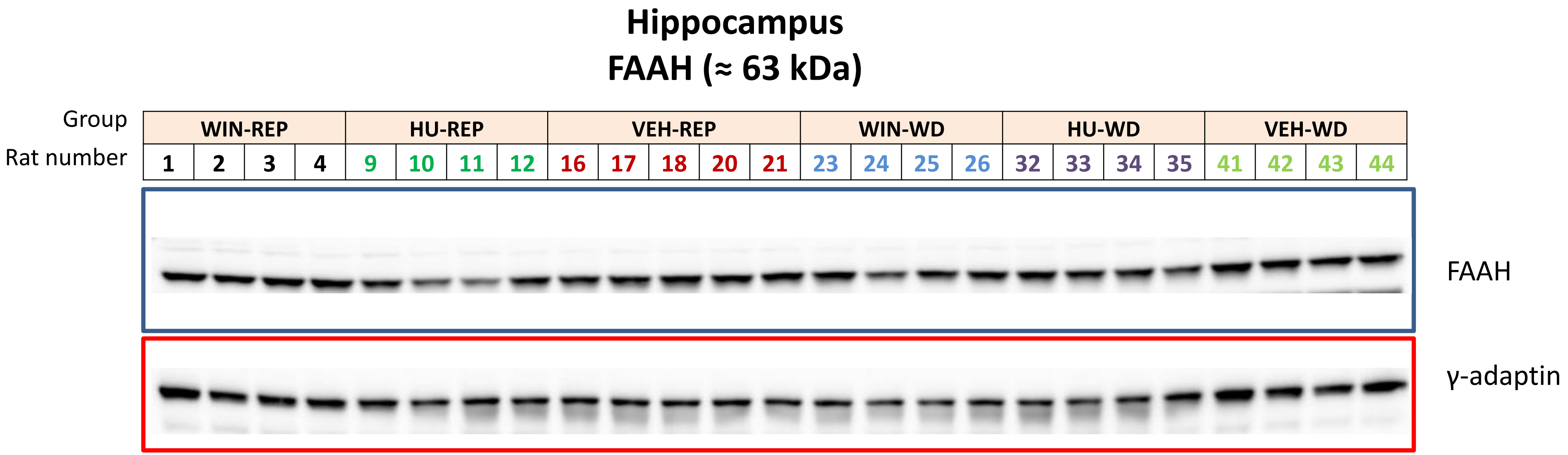

Gel 4: Ponceau S Red Staining

Hippocampus

Main components (receptors and enzymes) of the endocannabinoid system: DAGLβ, NAPE-PLD, MAGL

Group	MW (kDa)	WIN-REP				HU-REP				VEH-REP					WIN-WD				HU-WD				VEH-WD			
Rat number		1	2	3	4	9	10	11	12	16	17	18	20	21	23	24	25	26	32	33	34	35	41	42	43	44

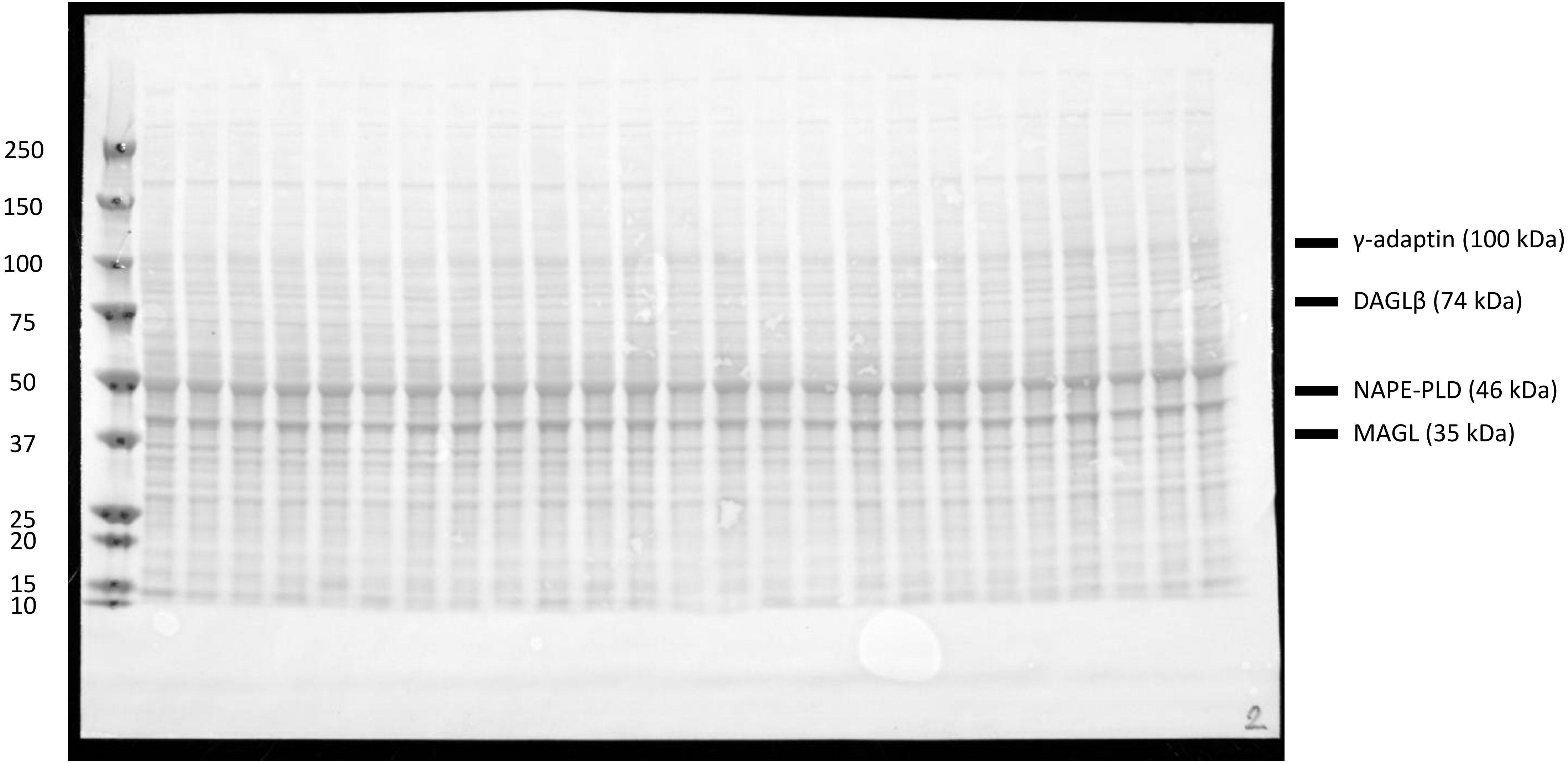

Membrane 4: Immunoblot

Hippocampus
DAGLβ (≈ 74 kDa)

Group	WIN-REP				HU-REP				VEH-REP					WIN-WD				HU-WD				VEH-WD			
Rat number	1	2	3	4	9	10	11	12	16	17	18	20	21	23	24	25	26	32	33	34	35	41	42	43	44

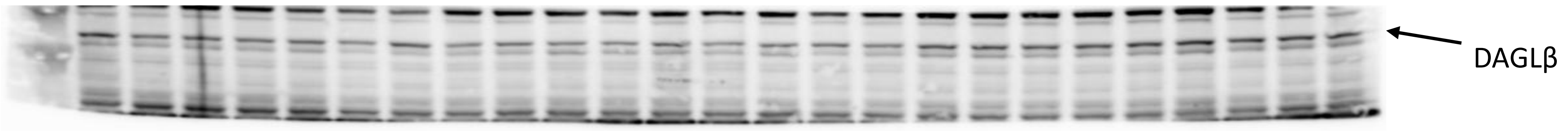

Membrane 4: Immunoblot

Hippocampus
DAGLβ (≈ 74 kDa)

Group	WIN-REP				HU-REP				VEH-REP					WIN-WD				HU-WD				VEH-WD			
Rat number	1	2	3	4	9	10	11	12	16	17	18	20	21	23	24	25	26	32	33	34	35	41	42	43	44

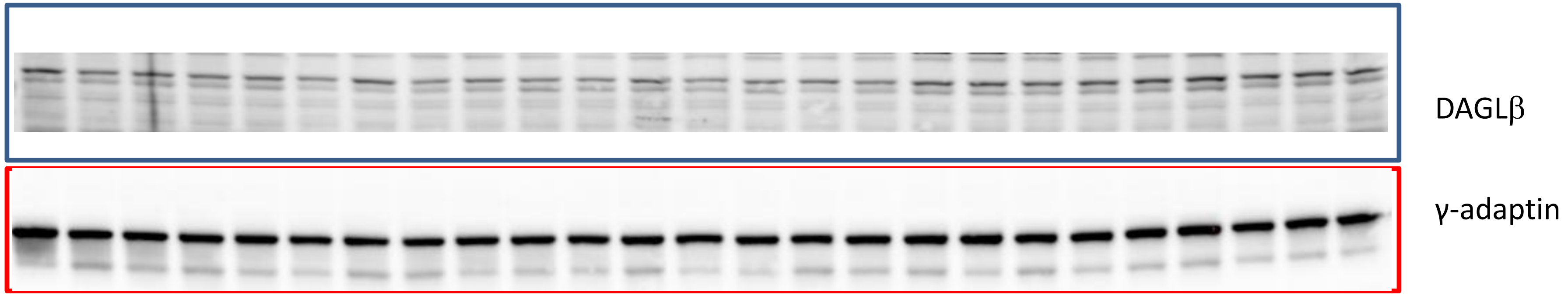

Membrane 4: Immunoblot

Hippocampus
MAGL (≈ 35 kDa)

Group	WIN-REP				HU-REP				VEH-REP					WIN-WD				HU-WD				VEH-WD			
Rat number	1	2	3	4	9	10	11	12	16	17	18	20	21	23	24	25	26	32	33	34	35	41	42	43	44

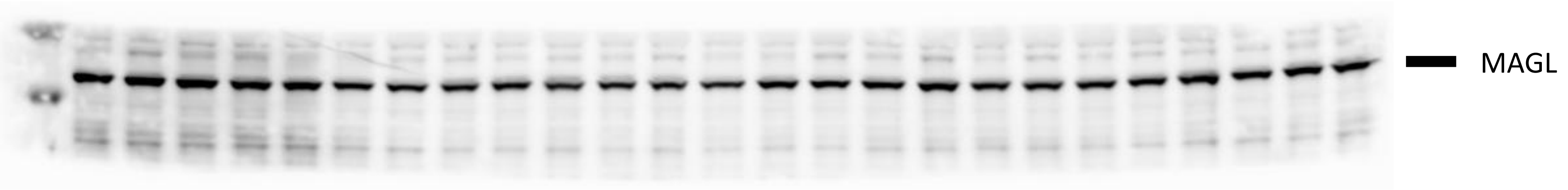

Membrane 4: Immunoblot

Hippocampus
MAGL (≈ 35 kDa)

Group	WIN-REP				HU-REP				VEH-REP					WIN-WD				HU-WD				VEH-WD			
Rat number	1	2	3	4	9	10	11	12	16	17	18	20	21	23	24	25	26	32	33	34	35	41	42	43	44

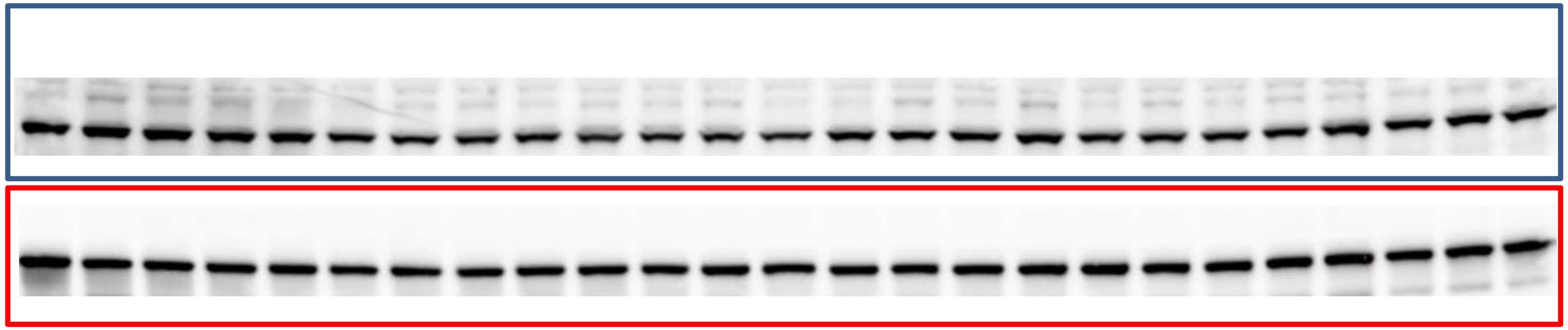

Membrane 4: Immunoblot

Hippocampus
NAPE-PLD (≈ 46 kDa)

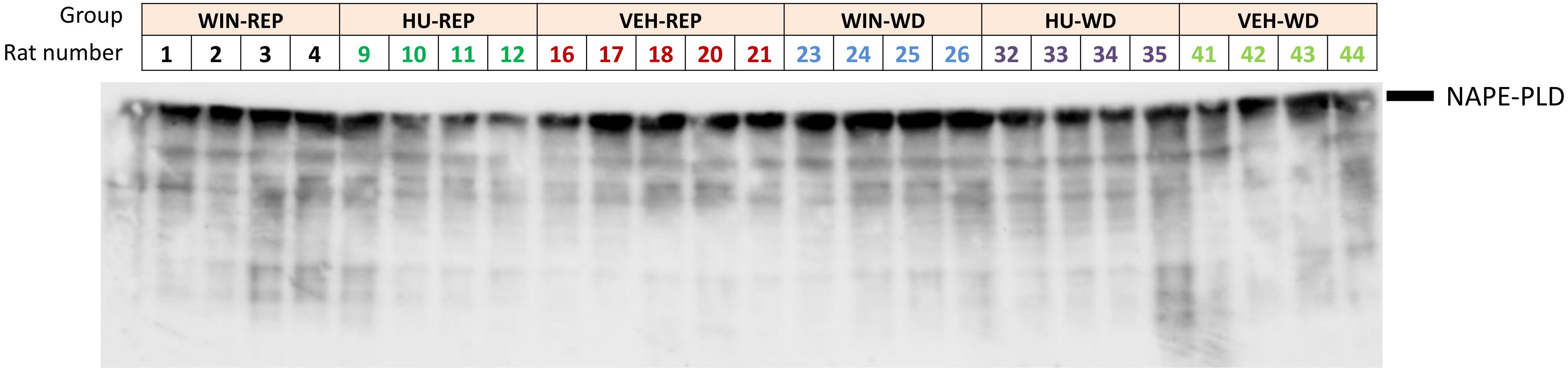

Membrane 4: Immunoblot

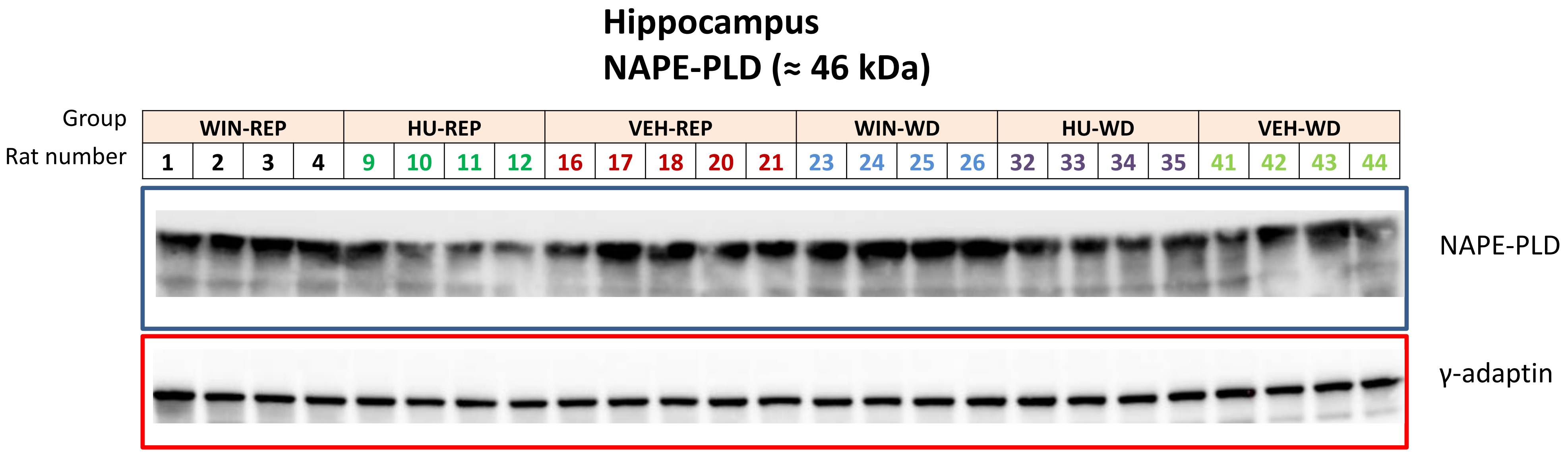

Gel 5: Ponceau S Red Staining

Hippocampus
Biomarkers of glial cells: GFAP, IBA1, vimentin

Group	MW (kDa)	WIN-REP				HU-REP				VEH-REP					WIN-WD				HU-WD				VEH-WD			
Rat number		1	2	3	4	9	10	11	12	16	17	18	20	21	23	24	25	26	32	33	34	35	41	42	43	44

missing

Membrane 5: Immunoblot

Hippocampus
GFAP (≈ 48 kDa)

Group	WIN-REP				HU-REP				VEH-REP					WIN-WD				HU-WD				VEH-WD			
Rat number	1	2	3	4	9	10	11	12	16	17	18	20	21	23	24	25	26	32	33	34	35	41	42	43	44

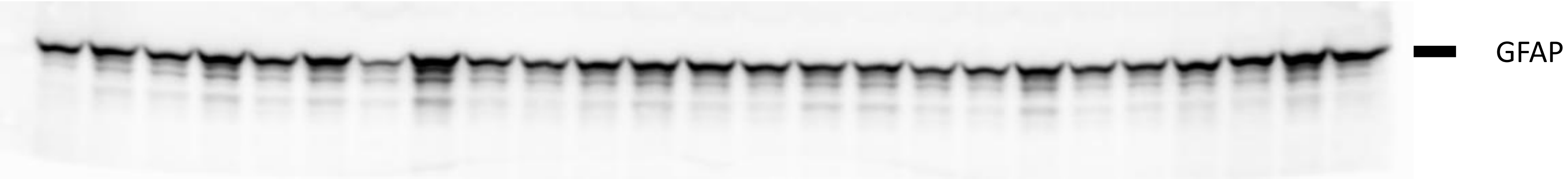

Membrane 5: Immunoblot

Hippocampus
GFAP (≈ 48 kDa)

Group	WIN-REP				HU-REP				VEH-REP					WIN-WD				HU-WD				VEH-WD			
Rat number	1	2	3	4	9	10	11	12	16	17	18	20	21	23	24	25	26	32	33	34	35	41	42	43	44

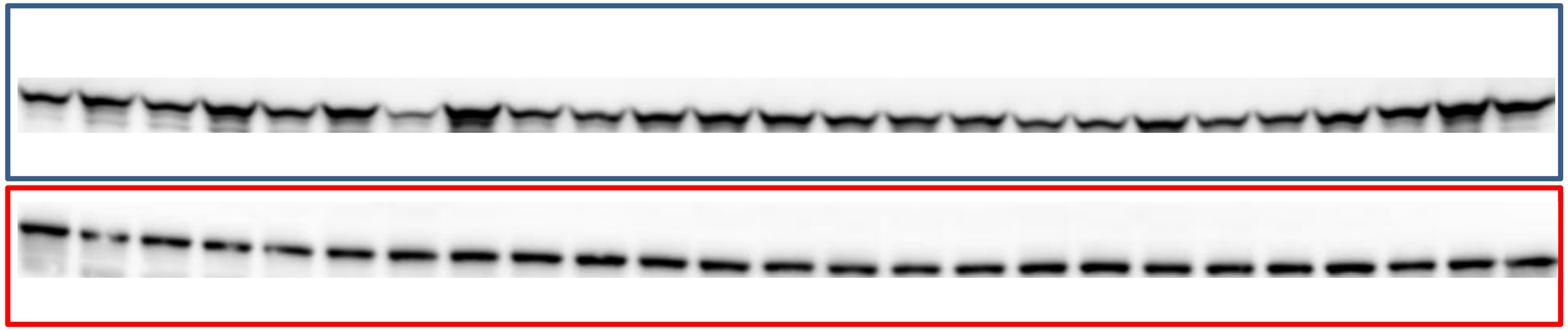

GFAP

γ-adaptin

Membrane 5: Immunoblot

Hippocampus
IBA1 (≈ 17 kDa)

Group	WIN-REP				HU-REP				VEH-REP					WIN-WD				HU-WD				VEH-WD			
Rat number	1	2	3	4	9	10	11	12	16	17	18	20	21	23	24	25	26	32	33	34	35	41	42	43	44

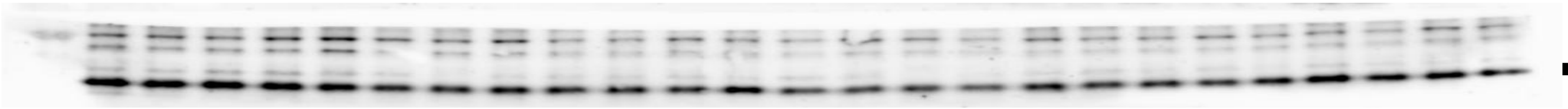

IBA1

Membrane 5: Immunoblot

Hippocampus
IBA1 (≈ 17 kDa)

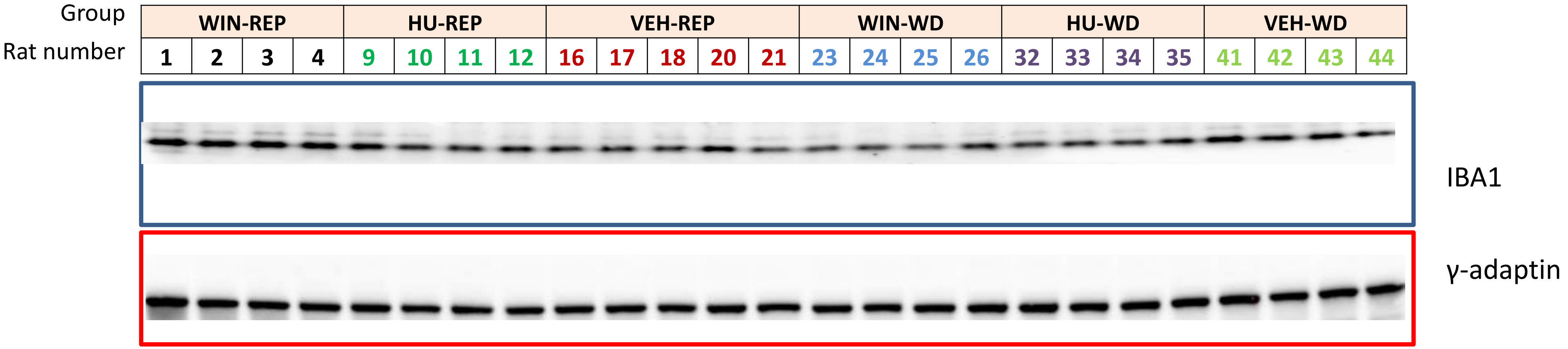

Membrane 5: Immunoblot

Hippocampus
Vimentin (≈ 54 kDa)

Group	WIN-REP				HU-REP				VEH-REP					WIN-WD				HU-WD				VEH-WD			
Rat number	1	2	3	4	9	10	11	12	16	17	18	20	21	23	24	25	26	32	33	34	35	41	42	43	44

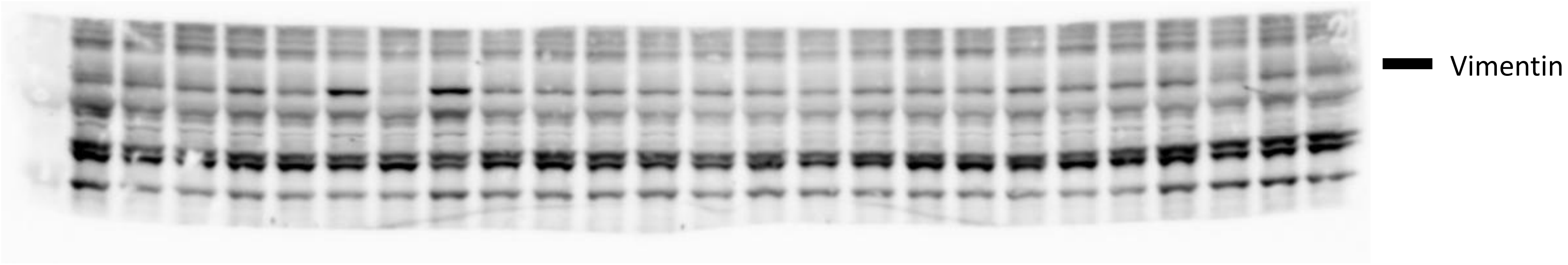

Membrane 5: Immunoblot

Hippocampus
Vimentin (≈ 54 kDa)

Group	WIN-REP				HU-REP				VEH-REP					WIN-WD				HU-WD				VEH-WD			
Rat number	1	2	3	4	9	10	11	12	16	17	18	20	21	23	24	25	26	32	33	34	35	41	42	43	44

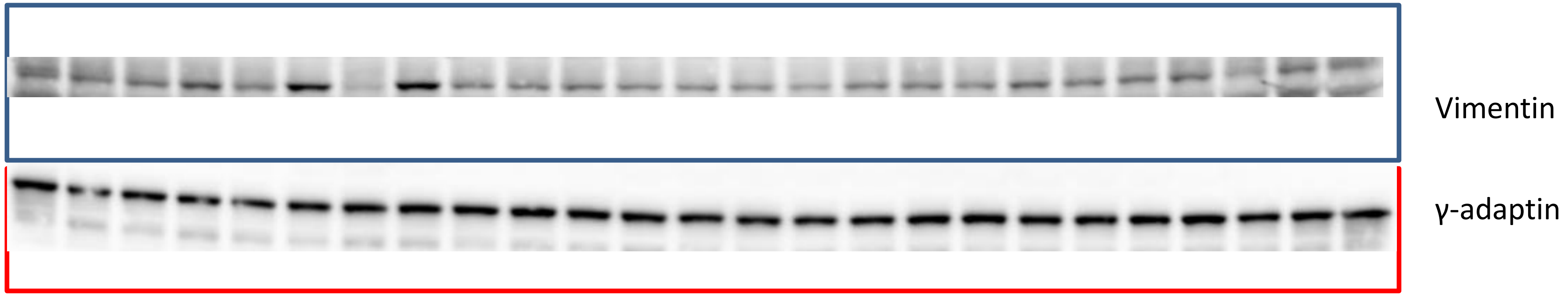

Gel 6: Ponceau S Red Staining

Hippocampus
Factors involved in neuroinflammation: COX2

Group	MW (kDa)	WIN-REP				HU-REP				VEH-REP					WIN-WD				HU-WD				VEH-WD			
Rat number		1	2	3	4	9	10	11	12	16	17	18	20	21	23	24	25	26	32	33	34	35	41	42	43	44

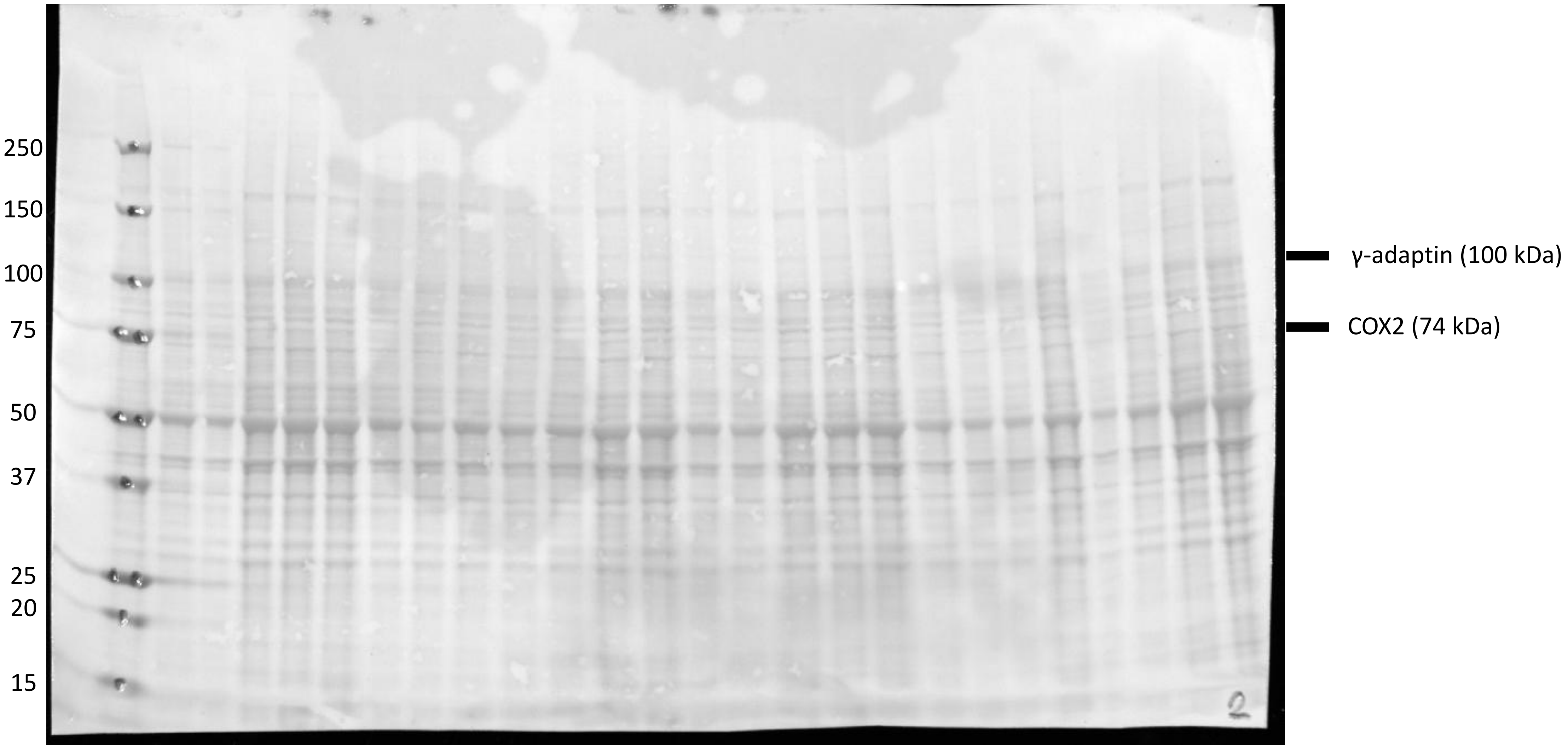

Membrane 6: Immunoblot

Hippocampus
COX2 (≈ 74 kDa)

Group	WIN-REP				HU-REP				VEH-REP					WIN-WD				HU-WD				VEH-WD			
Rat number	1	2	3	4	9	10	11	12	16	17	18	20	21	23	24	25	26	32	33	34	35	41	42	43	44

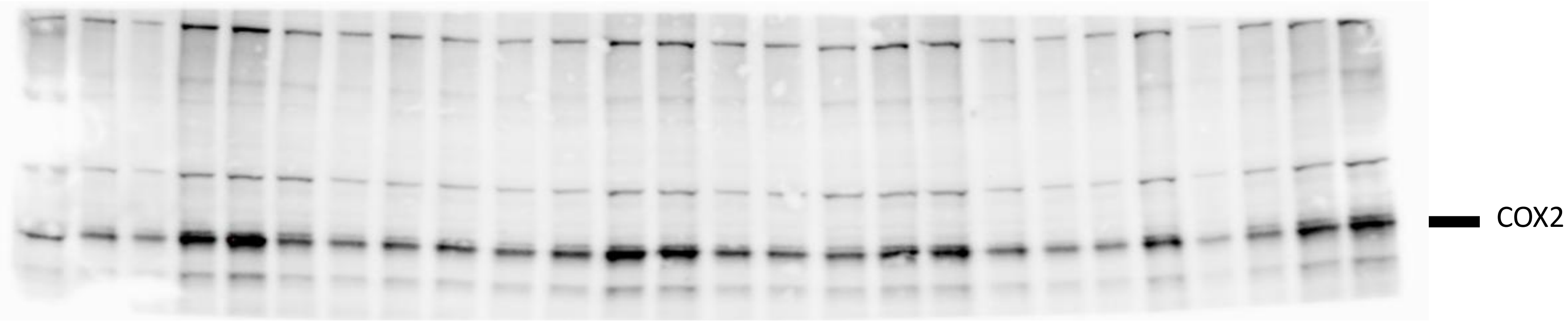

Membrane 6: Immunoblot

Hippocampus
COX2 (≈ 74 kDa)

Group	WIN-REP				HU-REP				VEH-REP					WIN-WD				HU-WD				VEH-WD			
Rat number	1	2	3	4	9	10	11	12	16	17	18	20	21	23	24	25	26	32	33	34	35	41	42	43	44

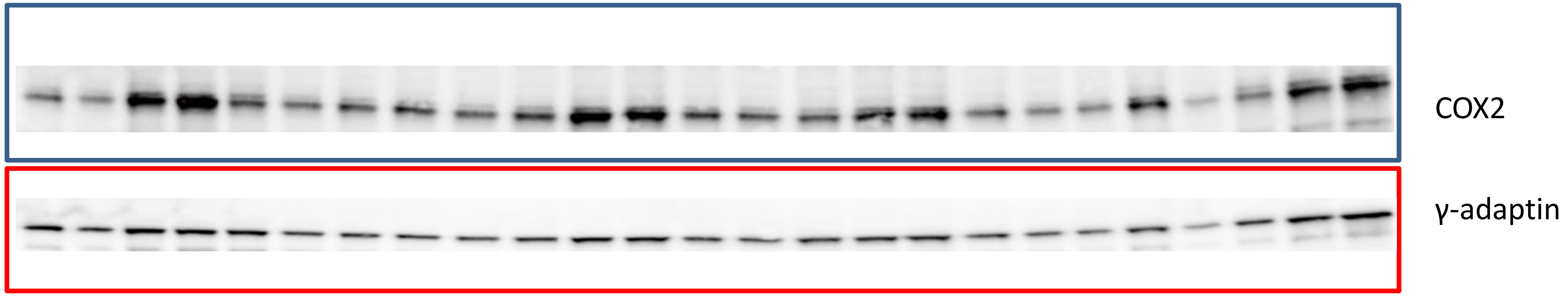

Gel 7: Ponceau S Red Staining

Hippocampus
Factors involved in neuroinflammation: IKKβ, NF-κB

Group	MW (kDa)	WIN-REP				HU-REP				VEH-REP					WIN-WD				HU-WD				VEH-WD			
Rat number		1	2	3	4	9	10	11	12	16	17	18	20	21	23	24	25	26	32	33	34	35	41	42	43	44

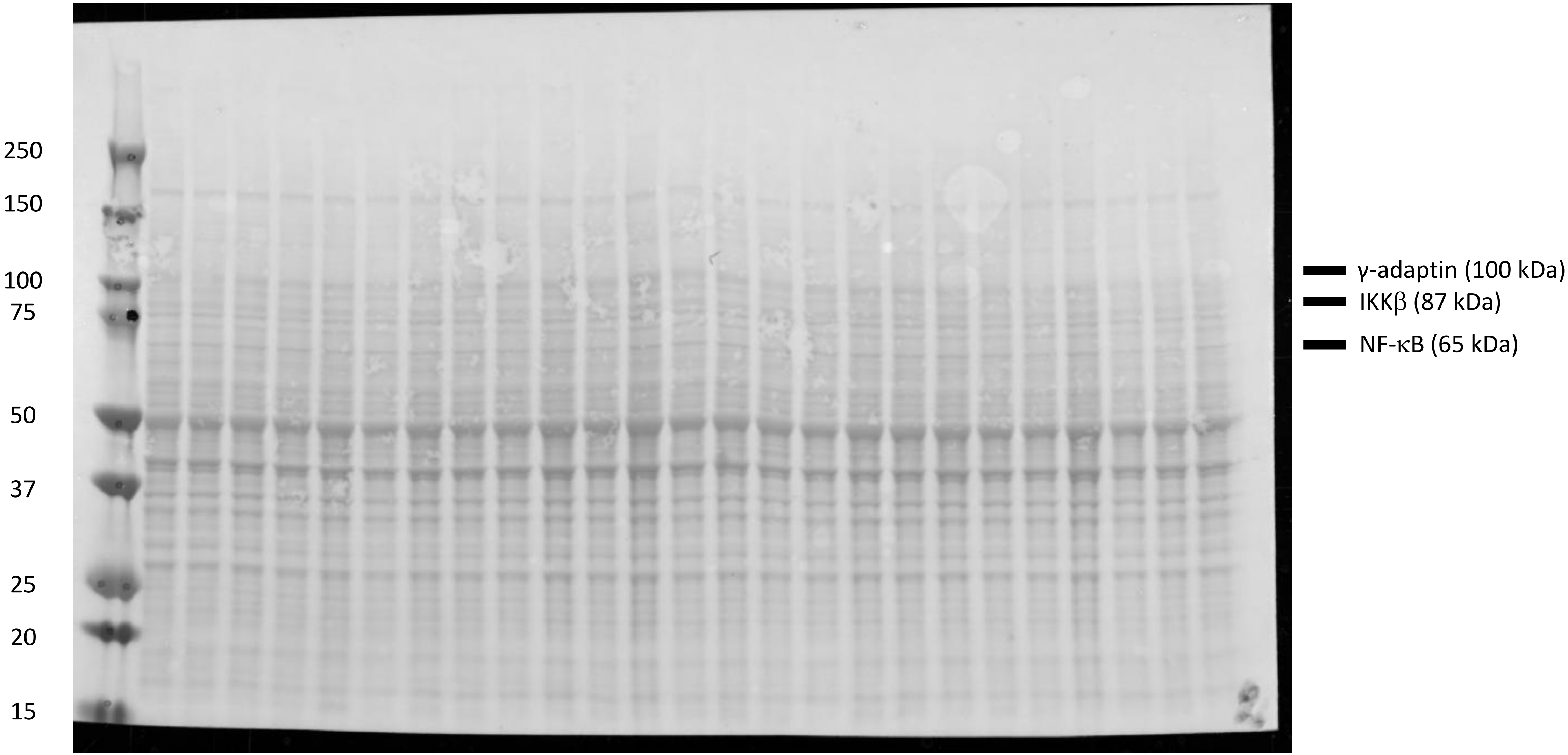

Membrane 7: Immunoblot

Hippocampus
NF-κB (≈ 65 kDa)

Group	WIN-REP				HU-REP				VEH-REP					WIN-WD				HU-WD				VEH-WD			
Rat number	1	2	3	4	9	10	11	12	16	17	18	20	21	23	24	25	26	32	33	34	35	41	42	43	44

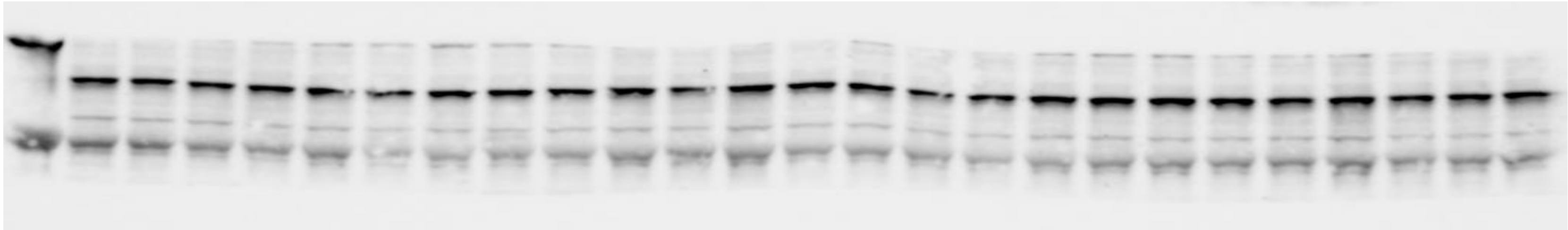

NF-κB

Membrane 7: Immunoblot

Hippocampus
NF-κB (≈ 65 kDa)

Group	WIN-REP				HU-REP				VEH-REP					WIN-WD				HU-WD				VEH-WD			
Rat number	1	2	3	4	9	10	11	12	16	17	18	20	21	23	24	25	26	32	33	34	35	41	42	43	44

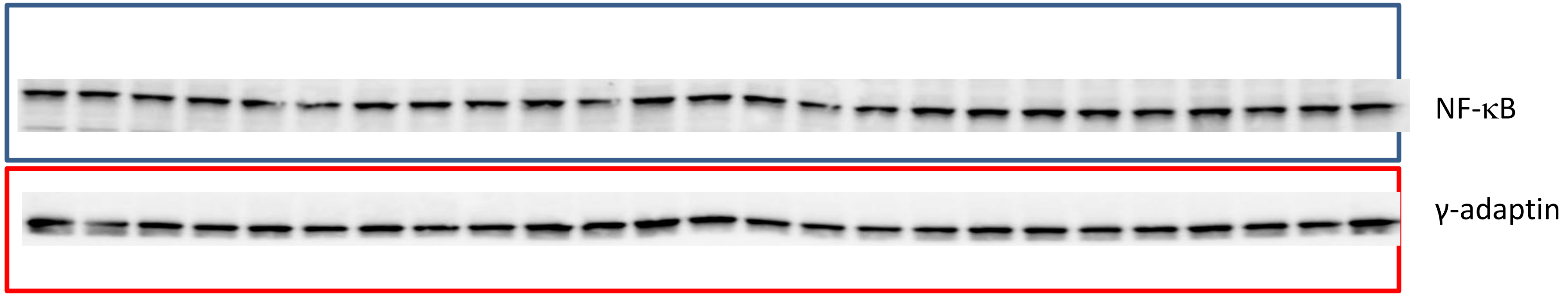

Membrane 7: Immunoblot

Hippocampus
IKKβ (≈ 87 kDa)

Group	WIN-REP				HU-REP				VEH-REP					WIN-WD				HU-WD				VEH-WD			
Rat number	1	2	3	4	9	10	11	12	16	17	18	20	21	23	24	25	26	32	33	34	35	41	42	43	44

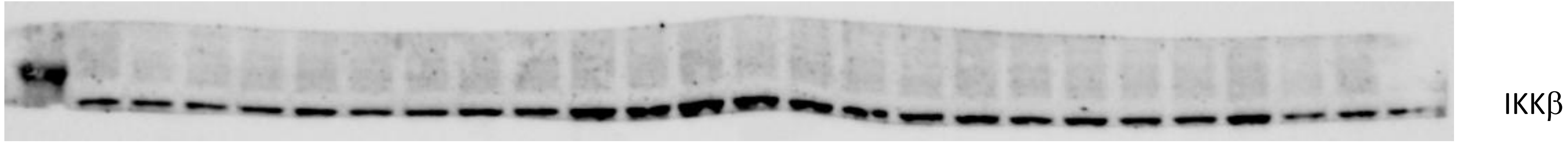

Membrane 7: Immunoblot

Hippocampus
IKKβ (≈ 87 kDa)

Group	WIN-REP				HU-REP				VEH-REP					WIN-WD				HU-WD				VEH-WD			
Rat number	1	2	3	4	9	10	11	12	16	17	18	20	21	23	24	25	26	32	33	34	35	41	42	43	44

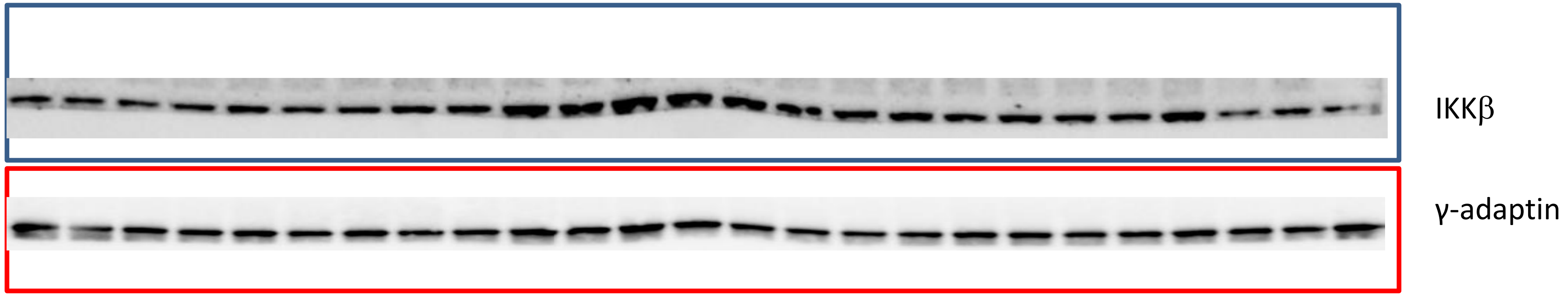

Summary of immunoblots (see Figure 4)

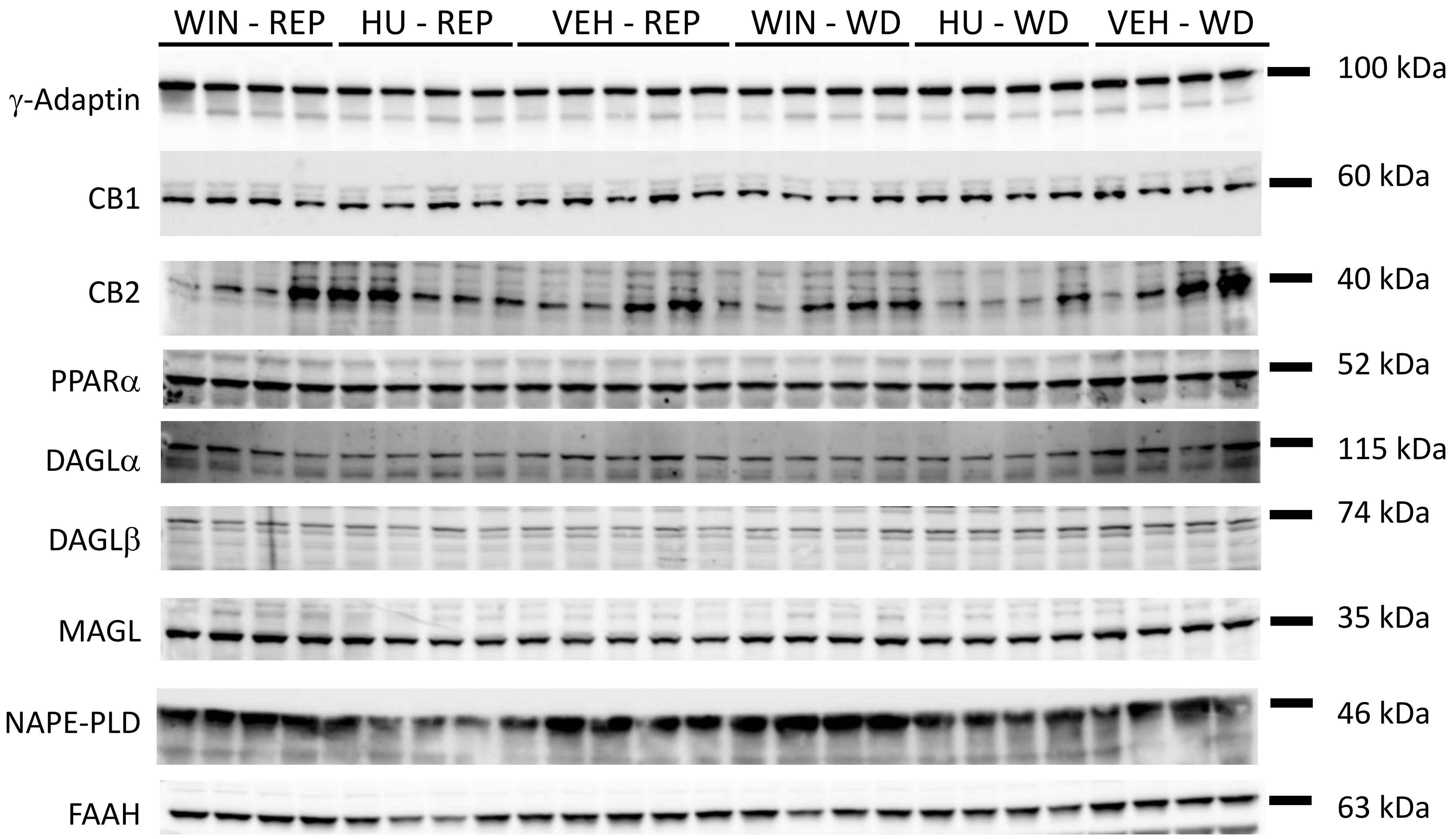

Summary of immunoblots (see Figure 5)

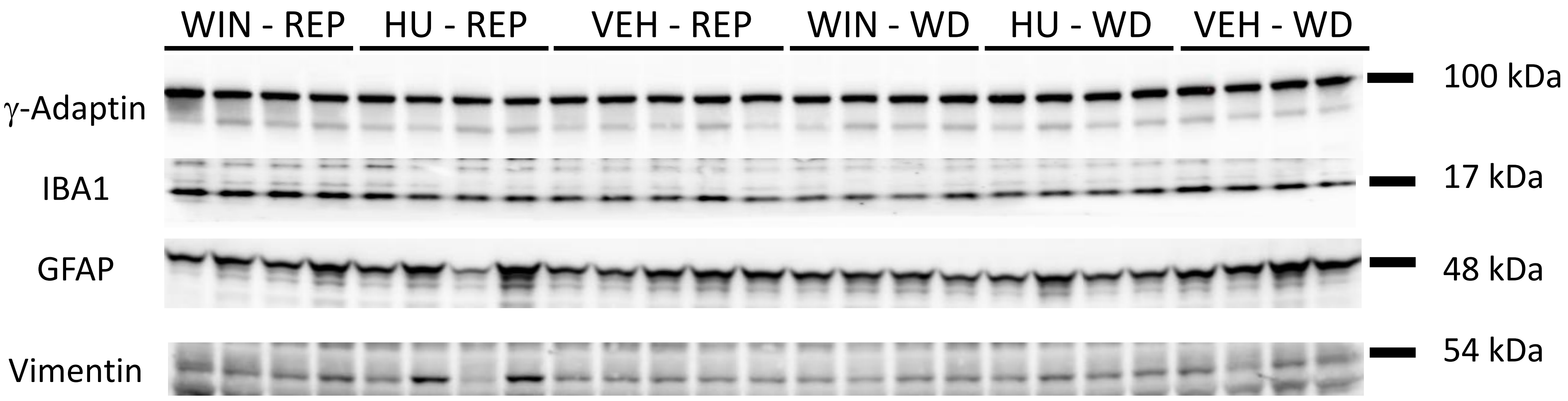

Summary of immunoblots (see Figure 5)

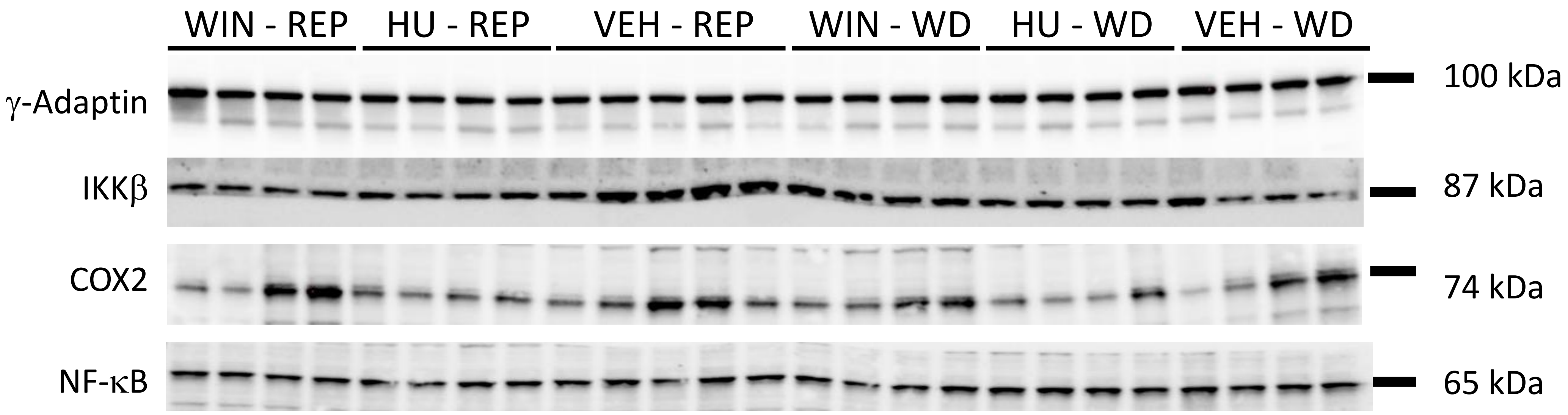

Supplement: Supplementary file 1 [file biomolecules-15-00417-s001.zip › biomolecules-3479269-supplementary.pdf]
